# Metabolite profiles reveal interspecific variation in operation of the Calvin–Benson cycle in both C_4_ and C_3_ plants

**DOI:** 10.1093/jxb/erz051

**Published:** 2019-02-18

**Authors:** Stéphanie Arrivault, Thiago Alexandre Moraes, Toshihiro Obata, David B Medeiros, Alisdair R Fernie, Alix Boulouis, Martha Ludwig, John E Lunn, Gian Luca Borghi, Armin Schlereth, Manuela Guenther, Mark Stitt

**Affiliations:** 1 Max Planck Institute of Molecular Plant Physiology, Am Muehlenberg, Potsdam-Golm, Germany; 2 School of Molecular Sciences, The University of Western Australia, Crawley WA, Australia

**Keywords:** C_4_, C_3_, Calvin–Benson cycle, interspecies variation, metabolite profiles, photosynthesis

## Abstract

Low atmospheric CO_2_ in recent geological time led to the evolution of carbon-concentrating mechanisms (CCMs) such as C_4_ photosynthesis in >65 terrestrial plant lineages. We know little about the impact of low CO_2_ on the Calvin–Benson cycle (CBC) in C_3_ species that did not evolve CCMs, representing >90% of terrestrial plant species. Metabolite profiling provides a top-down strategy to investigate the operational balance in a pathway. We profiled CBC intermediates in a panel of C_4_ (*Zea mays*, *Setaria viridis*, *Flaveria bidentis*, and *F. trinervia*) and C_3_ species (*Oryza sativa*, *Triticium aestivum*, *Arabidopsis thaliana*, *Nicotiana tabacum*, and *Manihot esculenta*). Principal component analysis revealed differences between C_4_ and C_3_ species that were driven by many metabolites, including lower ribulose 1,5-bisphosphate in C_4_ species. Strikingly, there was also considerable variation between C_3_ species. This was partly due to different chlorophyll and protein contents, but mainly to differences in relative levels of metabolites. Correlation analysis indicated that one contributory factor was the balance between fructose-1,6-bisphosphatase, sedoheptulose-1,7-bisphosphatase, phosphoribulokinase, and Rubisco. Our results point to the CBC having experienced different evolutionary trajectories in C_3_ species since the ancestors of modern plant lineages diverged. They underline the need to understand CBC operation in a wide range of species.

## Teaser

Interspecific divergence in metabolite profiles in C_3_ and C_4_ species points to differing evolutionary trajectories of the Calvin–Benson cycle in different seed plant lineages

## Introduction

The Calvin–Benson cycle (CBC) evolved ~2 billion years ago (Rasmussen *et al.*, 2008), is the most abundant biochemical pathway on Earth in terms of nitrogen investment (Ellis, 1979; Raven, 2013), and plays a dominant role in the global carbon (C) and O_2_ cycles. The CBC can be divided into three partial processes; fixation of CO_2_ (ribulose-1,5-bisphosphate carboxylase-oxygenase) RuBisCO into a 3-C compound, 3-phosphoglycerate (3PGA), reduction of 3PGA to triose phosphate (triose-P) using ATP and NADPH from the light reactions, and a series of reactions that use triose-P to regenerate ribulose 1,5-bisphosphate (RuBP) (von Caemmerer and Farquhar, 1981; Heldt, 2005; Stitt *et al.*, 2010; Adam, 2017). The net gain in C exits the CBC and is converted into end-products. Despite its evolutionary age, the pathway’s structure is essentially unchanged from cyanobacteria to angiosperms.

This conservation of the CBC pathway structure is remarkable. The CBC evolved in a world in which CO_2_ concentrations were very high and O_2_ concentrations were very low. Over geological time, there has been a dramatic rise in atmospheric O_2_ and decline in atmospheric CO_2_. This uncovered a side reaction with O_2_, which competes with CO_2_ as a substrate for RuBisCO, leading to the formation of 2-phosphoglycolate (2PG) (Lorimer and Andrews, 1973; Lorimer, 1981; Tcherkez *et al.*, 2006). 2PG is recycled via an energetically wasteful process termed photorespiration that results in the loss of 0.5 CO_2_ per scavenged molecule of 2PG (Somerville, 2001; Heldt, 2005). In the current atmosphere with 0.04% CO_2_ and 21% O_2_, in C_3_ plants about every fourth reaction is with O_2_ instead of CO_2_, leading to a 20–30% decrease in the net rate of photosynthesis (Osmond, 1981; Sharkey, 1988; Long *et al.*, 2006; Betti *et al.*, 2016). This side reaction decreases nitrogen use efficiency, because higher amounts of protein must be invested in the photosynthetic apparatus. This includes an especially large investment in RuBisCO, which has a relatively low catalytic rate and represents up to half of leaf protein (Ellis, 1979; Betti *et al.*, 2016). It negatively impacts water use efficiency because a higher internal CO_2_ concentration is required to support a given net rate of photosynthesis, which in turn requires higher stomatal conductance and higher evaporative water loss (Ort *et al.*, 2015; Betti *et al.*, 2016).

Cyanobacteria and eukaryotic algae possess C-concentrating mechanisms (CCMs) that accumulate CO_2_ in RuBisCO-containing microstructures, the carboxysome in cyanobacteria and the pyrenoid in eukaryotic algae (Badger *et al.*, 1998; Giordano *et al.*, 2005; Kerfeld and Melnicki, 2016; Raven *et al.*, 2017). These microstructures were lost in plant lineages that colonized the land. A second type of CCM evolved in terrestrial plants in the last 30 million years (Sage *et al.*, 2012; Raven *et al.*, 2017), coinciding with the decline of CO_2_ from ~1000 ppm to <300 ppm during the Oligocene (Christin *et al.*, 2008; Zachos *et al.*, 2008; Edwards *et al.*, 2010). These CCMs are in essence biochemical CO_2_ pumps, in which bicarbonate is fixed into 4-C acids that are subsequently decarboxylated to generate a high internal CO_2_ concentration. In C_4_ plants, bicarbonate is typically captured by phosph*enol*pyruvate (PEP) carboxylase in mesophyll cells, and 4-C acids diffuse to bundle sheath cells, which are located internally within the leaf and contain RuBisCO and the rest of the CBC (Hatch, 2002; von Caemmerer and Furbank, 2003; Sage *et al.*, 2012; Sage, 2017). There is substantial diversity in the pathway of C_4_ photosynthesis; for example, which 4-C and 3-C metabolites are involved in the CCM, how the 4-C acid is decarboxylated, and to what extent PSII activity is lost in the bundle sheath chloroplasts. C_4_ photosynthesis evolved independently >65 times in separate lineages among the angiosperms, and C_4_ species currently represent ~3% of terrestrial plant species and account for 23% of total terrestrial C gain (Still *et al.*, 2003; Sage *et al.*, 2011; Sage, 2017). An analogous biochemical CO_2_ pump evolved in plants with Crassulacean acid metabolism (CAM); bicarbonate is assimilated in the dark into 4-C acids, which are decarboxylated in the light to provide CO_2_ for the CBC (Shameer *et al.*, 2018). CAM evolved in at least 35 independent lineages and is found in ~6% of current terrestrial plant species (Silvera *et al.*, 2010). Parallel evolution of C_4_ and CAM in many lineages underlines the strong selective pressure exerted by low CO_2_ in the recent geological past.

CCMs are complex traits. For example, C_4_ photosynthesis requires major changes in leaf development and anatomy, gene expression patterns, and the location, levels, and properties of hundreds of enzymes and transporters (Sage *et al.*, 2012; Heckmann *et al.*, 2013; Sage, 2017). It is likely that its evolution involved successive steps, including the development of denser venation, modification of the size and functionality of bundle sheath cells, and stepwise specialization of metabolism in the bundle sheath and mesophyll cells (McKown and Dengler, 2007; Kocacinar *et al.*, 2008; Nelson, 2011; Sage *et al.*, 2013; Mallmann *et al.*, 2014). This multistep evolutionary trajectory may explain why CCMs evolved in only a relatively small fraction of terrestrial plant lineages (Heckmann, 2016).

Low CO_2_ will have exerted massive selective pressure on the CBC in species that did not evolve a CCM, representing ~90% of existing terrestrial plant species (Silvera *et al.*, 2010; Sage, 2017). Pressure will also have been exerted by other environmental factors such as water availability, temperature, and nutrient availability (Raven *et al.*, 2017). Indeed, terrestrial C_3_ plants exhibit substantial variation in photosynthetic rate, with large differences between annuals and perennials, and considerable differences within these groups (Evans, 1989; Wullschleger, 1993). This includes variation in photosynthetic rate between phylogenetically related species (Galmés *et al*., 2014*b*) and within species (Driever *et al.*, 2014). Factors contributing to variation in photosynthetic rate include differences in the rate of electron transport and carboxylation (Wullschleger, 1993), leaf nitrogen content and photosynthetic nitrogen use efficiency (Field and Mooney, 1986; Evans, 1989; Hikosaka, 2010), and differing investment strategies in short-lived (deciduous) and long-lived (evergreen) leaves (Wright *et al.*, 2004; Donovan *et al.*, 2011).

We know relatively little about whether there is interspecific variation in the CBC in C_3_ plants (Lawson *et al.*, 2012). It is well established that RuBisCO kinetics have evolved over a long geological time scale, with selectivity for CO_2_ rising and catalytic rate declining between cyanobacteria and higher plants (Jordan and Ogren, 1981; Badger *et al.*, 1998; Tcherkez *et al.*, 2006; Savir *et al.*, 2010; Sharwood *et al.*, 2016a, b). Intriguingly, there is also variance over shorter evolutionary time scales. RuBisCO kinetics vary between quite closely related C_3_ species (Yeoh *et al.*, 1980; Galmés *et al*., 2014*a*; Prins *et al.*, 2016). In perennial oak, ecological adaptations have been linked to specific amino acid polymorphisms in RuBisCO (Hermida-Carrera *et al.*, 2017). RuBisCO is inhibited by RuBP and low molecular weight inhibitors that derive from catalytic infidelities of RuBisCO or, like 2-carboxyarabinitol 1-phosphate, are synthesized by other enzymes (Yeoh *et al.*, 1980; Parry *et al.*, 2008). There is surprising diversity in the levels and dynamics of these low molecular weight inhibitors in different C_3_ species (Servaites *et al.*, 1986; Moore *et al.*, 1993; Charlet *et al.*, 1997; Parry *et al.*, 2008) and, incidentally, different C_4_ species (Carmo-Silva *et al.*, 2010). CP12 is a small regulatory protein that interacts with NADP-glyceraldehyde-3-phosphate dehydrogenase (NADP-GAPDH) and phosphoribulokinase (PRK) (Gontero and Maberly, 2012; López-Calcagno *et al*., 2014). The action of CP12 varies between C_3_ species (Howard *et al.*, 2011; López-Calcagno *et al*., 2014), again pointing to interspecies variation in CBC regulation.

Some of the strongest evidence that the CBC can adapt to selection or relaxation of selection in a relatively short evolutionary time comes from studies of C_4_ species. Compared with C_3_ species, C_4_ species contain forms of RuBisCO with a lower affinity for CO_2_ and faster catalytic turnover (Yeoh *et al.*, 1980; Sage and Seemann, 1993; Kapralov *et al.*, 2011; Galmés *et al*., 2014*b*; Sharwood *et al.*, 2016a, b), allowing a substantial decrease in RuBisCO abundance (Long, 1999; Ghannoum *et al.*, 2005; Sharwood *et al.*, 2016*a*, *b*, *c*). Such changes are found even within the tribe Paniceae in which C_4_ photosynthesis evolved recently (Sharwood *et al.*, 2016*a*).

The operation of a pathway depends on many factors, including the abundance of the participating enzymes, their kinetic properties, and the action of regulatory mechanisms on individual enzymes and sets of enzymes. It is laborious to characterize variation in all these potential factors. Analyses of steady-state metabolite levels provide a top-down strategy to search for variation in pathway operation. This is because changes in enzyme abundance, properties, or regulation will all lead to changes in the relative levels of the metabolic intermediates in a pathway.

Information about CBC intermediate levels in different C_3_ species is rather sparse. Most previous studies in C_3_ plants focused on RuBP (e.g. Sage and Seemann, 1993) or a handful of metabolites such as 3PGA, triose-P, and fructose 1,6-bisphosphate (FBP), and were restricted to single species (see Stitt *et al.*, 2010 for references). A similar picture holds for C_4_ plants (Stitt and Heldt, 1985; Usuda, 1987; Leegood and von Caemmerer, 1988, 1989). The reason was partly conceptual, reflecting the idea that photosynthesis is usually limited by the light reactions or RuBisCO (Farquhar *et al.*, 1980). Subsequent work has highlighted that photosynthesis can also be limited by reactions in the remainder of the CBC (see Stitt *et al.*, 2010 for a review), especially sedoheptulose-1,7-bisphosphatase (SBPase) (Raines *et al.*, 2000; Lefebvre *et al.*, 2005; Zhu *et al.*, 2007; Ding *et al.*, 2016; Driever *et al.*, 2017; Simkin *et al.*, 2017). There were also technical reasons; until ~10 years ago it was impossible to quantify many CBC intermediates routinely. This is now possible using HPLC-MS/MS (Cruz *et al.*, 2008; Arrivault *et al.*, 2009; Hasunuma *et al.*, 2010; Ma *et al.*, 2014).

In this study, we have profiled CBC intermediates in four C_4_ species and five C_3_ species, representing diverse plant lineages including eudicots and monocots. We used these data to address two questions. The first is whether CBC intermediates display different profiles in C_3_ and C_4_ species, as would be expected if the presence of a CCM allows a different mode of CBC operation. This question provides a check that expected differences in CBC operation can be detected as changes in CBC metabolite profiles. In particular, we might expect that the lower abundance of RuBisCO (see above) results in lower levels of RuBP. Furthermore, C_4_ species with dimorphic chloroplasts might have enhanced levels of 3PGA and triose-P to support an intercellular shuttle that transfers energy from the mesophyll to the bundle sheath cells. The second and major question is whether there are interspecific differences between C_3_ species. This would have important implications for the evolution of the CBC and the need for a better understanding of the pathway in a broader range of C_3_ species, including many of our major crops.

## Materials and methods

### Chemicals

Carbon dioxide (^13^CO_2_, isotopic purity 99 atom%) was from Campro Scientific GmbH (Berlin, Germany; www.campro.eu), N_2_, O_2_, and unlabelled CO_2_ from Air Liquide (Germany; https://industrie.airliquide.de/), and chemicals were obtained from Sigma-Aldrich (Darmstadt, Germany; www.sigmaaldrich.com), Roche Applied Science (Mannheim, Germany; lifescience.roche.com), or Merck (www.merckmillipore.com).

### Plant growth and harvest

Nine species (of which eight were phylogenetically diverse; Supplementary Fig. S1 at *JXB* online) were grown as described in Supplementary Table S1. Material was harvested by cutting leaves and quenching them immediately in a bath of liquid N_2_ under growth irradiance, avoiding shading.

### Metabolite analyses

Plant material was ground to a fine powder by hand in a mortar pre-cooled with liquid N_2_ or in a cryo-robot (Stitt *et al.*, 2007) and stored at –80 °C. Metabolites were extracted and quantified by LC-MS/MS (Arrivault *et al.*, 2009). All samples were spiked with stable isotope-labelled internal standards for correction of ion suppression and other matrix effects (Arrivault *et al.*, 2015). 3PGA gives a broad, poorly defined peak in LC-MS/MS and was therefore quantified enzymatically (Merlo *et al.*, 1993).

### Chlorophyll and protein

Chl *a* and *b* were extracted and quantified as in Gibon *et al.* (2002). Protein was extracted from 20 mg FW ground plant material in 750 µl of buffer [0.1 M Tris–HCl, pH 8, 0.2 M NaCl, 5 mM EDTA, 2% (w/v) SDS, 0.2% (v/v) β-mercaptoethanol, and protease inhibitor cocktail (P9599, Sigma, Germany)]. The suspension was mixed well, incubated (30 min, room temperature), re-mixed, centrifuged (10 min, 1500 *g*, 4 °C), and the supernatant collected. Supernatants were pooled from two or (*Oryza sativa* and *Manihot esculenta*) three successive extractions. Protein was quantified colorimetrically with bicinchoninic acid (BCA Protein Assay-Reducing Agent Compatible, Thermo Fisher Scientific, Germany; www.thermofisher.com) with BSA as standard.

### Gas exchange

CO_2_ assimilation was measured using the fourth fully expanded *Zea mays* leaf or 5-week-old *Arabidopsis thaliana* rosettes using an open-flow infrared gas exchange analyser system (LI-COR Inc., Lincoln, NE, USA; www.licor.com) equipped with an integrated fluorescence chamber head (LI-6400-40, 2 cm^2^ leaf chamber for *Z. mays*; LI-6400-17 whole-plant Arabidopsis chamber for *A. thaliana*; LI-COR Inc.). CO_2_ was kept at 400 µmol mol^–1^, leaf temperature at 29 °C for *Z. mays* and at 20 °C for *A. thaliana*, and relative humidity at 65%.

### ^13^CO_2_labelling with *M. esculenta*

The fifth or sixth fully expanded leaf from the top of a 9-week-old plant was labelled (Supplementary Fig. S2A), starting 2 h into the light period. The leaf was placed in the labelling chamber (Supplementary Fig. S2B, C; see Arrivault *et al.*, 2017). Gases were supplied from individual bottles and controlled by gas-flow controllers (Brooks instruments; www.brooksinstrument.com). The labelling chamber was initially supplied with 79% N_2_, 21% O_2_, and 420 ppm ^12^CO_2_. After 1 min, ^12^CO_2_ was replaced by ^13^CO_2_. Samples were collected after 10, 20, 40, or 60 s, or 2, 5, 10, 30, or 60 min, in random order. Gas flow was 10 l min^−1^ for pulses of up to 1 min, and 5 l min^−1^ for longer pulses. Unlabelled samples (*t*=0) were collected after 1 min in unlabelled gas mixture. The chamber was maintained at growth cabinet temperature (28 °C) by circulating water from a water bath. Gases were passed through a humidifier in the water bath after mixing and before entering the measuring chamber. Light intensity at the leaf surface was kept as in the growth cabinet (250 µmol m^−2^ s^−1^) by supplying additional light (FL-460 Lighting Unit, Walz, Effeltrich, Germany). Material was quenched by dropping a copper rod, pre-cooled in liquid N_2_, down a hollow tube incorporated in the chamber lid, thereby freeze-clamping a 1.9 cm diameter (~40 mg FW) leaf disc (Supplementary Fig. S2C, D). ^13^CO_2_-labelled samples were analysed by LC-MS/MS and GC-MS, and isotopomer distribution (%) and enrichment (%) were calculated as in Arrivault *et al.* (2017).

### Statistical analyses

Statistical analysis was performed in R Studio Version 0.99.896 (www.rstudio.com) with R version 3.3.0 (https://cran.r-project.org/) using either Student’s *t*-test (R default package stats) or an ANOVA (Sums of Squares Type II) followed by the Tukey’s Honest Significant Differences (HSD) post-test (R package agricolae). Details are provided in the figure legends.

## Results

### Metabolite levels at growth irradiance

We profiled CBC metabolites in four C_4_ species from the NADP-malic enzyme subtype including two monocots (*Zea mays* and *Setaria viridis*) and two eudicots (*Flaveria bidentis* and *F. trinervia*), and five C_3_ species including two monocots (*Oryza sativa*, *Triticium aestivum*) and three eudicots (*Arabidopsis thaliana*, *Nicotiana tabacum*, and *Manihot esculenta*). Each species was grown with non-saturating irradiance (range of 60–133% of that required for half-maximal rates of photosynthesis) and appropriate temperature for rapid, healthy growth, and harvested under growth irradiance at least 2 h after the beginning of the light period (for details, see Supplementary Table S1). CBC intermediates and 2PG levels were determined by LC-MS/MS, using isotope-labelled internal standards to obtain reliable quantification, or enzymatically (3PGA). The signals for ribulose-5-phosphate (Ru5P) and xylulose-5-phosphate (Xu5P) overlapped, so they were combined (‘Ru5P+Xu5P’). Otherwise, we were able to quantify all CBC intermediates except 1,3-bisphosphoglycerate, glyceraldehyde 3-phosphate, and erythrose 4-phosphate. Metabolites were initially normalized on FW.

**Fig. 1.**
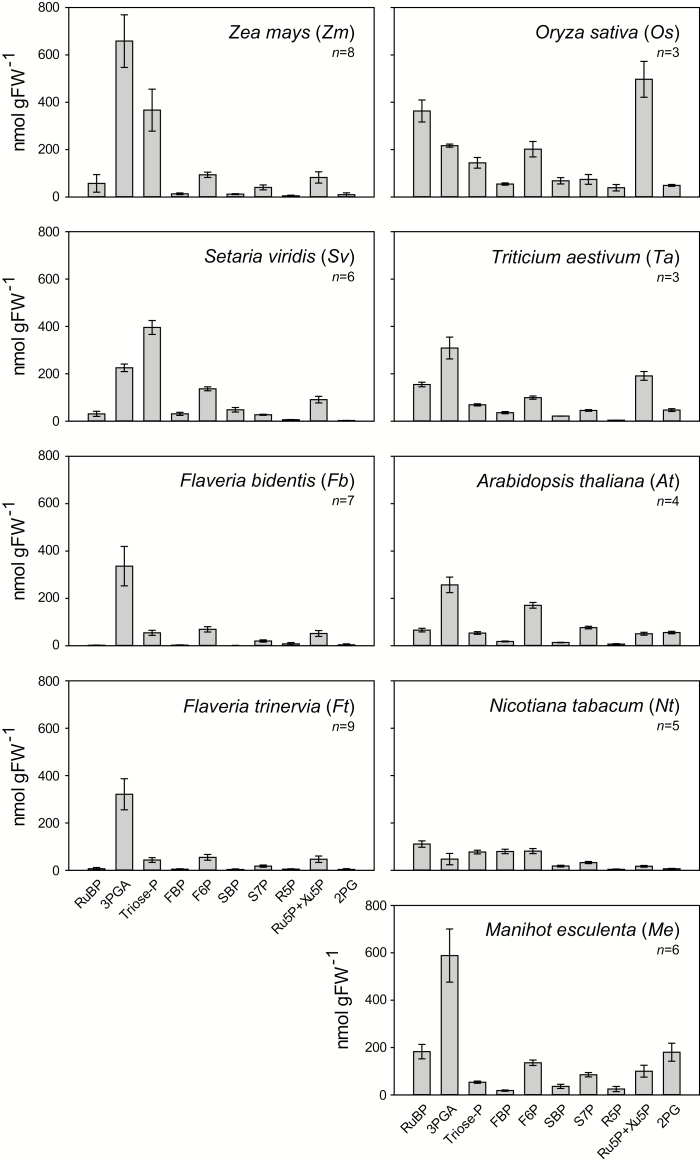
CBC metabolite and 2PG profiles in different species. Growth and harvest conditions can be found in Supplementary Table S1. Note that 2PG amounts are multiplied by 10 for better visibility. The results are shown as mean (nmol g FW^−1^) ±SD. The original data are provided in Supplementary Dataset S1.

CBC metabolite levels varied greatly between species (Fig. 1; Supplementary Dataset S1). This involved differences in the absolute and the relative levels of metabolites. Some of the observed changes were expected, for example the low levels of 2PG in C_4_ compared with C_3_ species, reflecting the lower rate of photorespiration in the C_4_ plants (note, 2PG amounts are multiplied by 10 for better visualization in Fig. 1). RuBP levels were lower in C_4_ compared with C_3_ species, probably reflecting lower abundance of RuBisCO in C_4_ plants. However, other interspecies differences were unexpected, in particular the rather diverse profiles in the five C_3_ species. Features that varied between the C_3_ species included the absolute levels of individual metabolites such as 3PGA, triose-P, Ru5P+Xu5P, the level of RuBP compared with metabolites involved in RuBP regeneration, and the relative levels of metabolite pairs, for example FBP and fructose 6-phosphate (F6P) or sedoheptulose 1,7-bisphosphate (SBP) and sedoheptulose 7-phosphate (S7P).

### Metabolite levels in *Z. mays* and *A. thaliana* at different irradiances

**Fig. 2.**
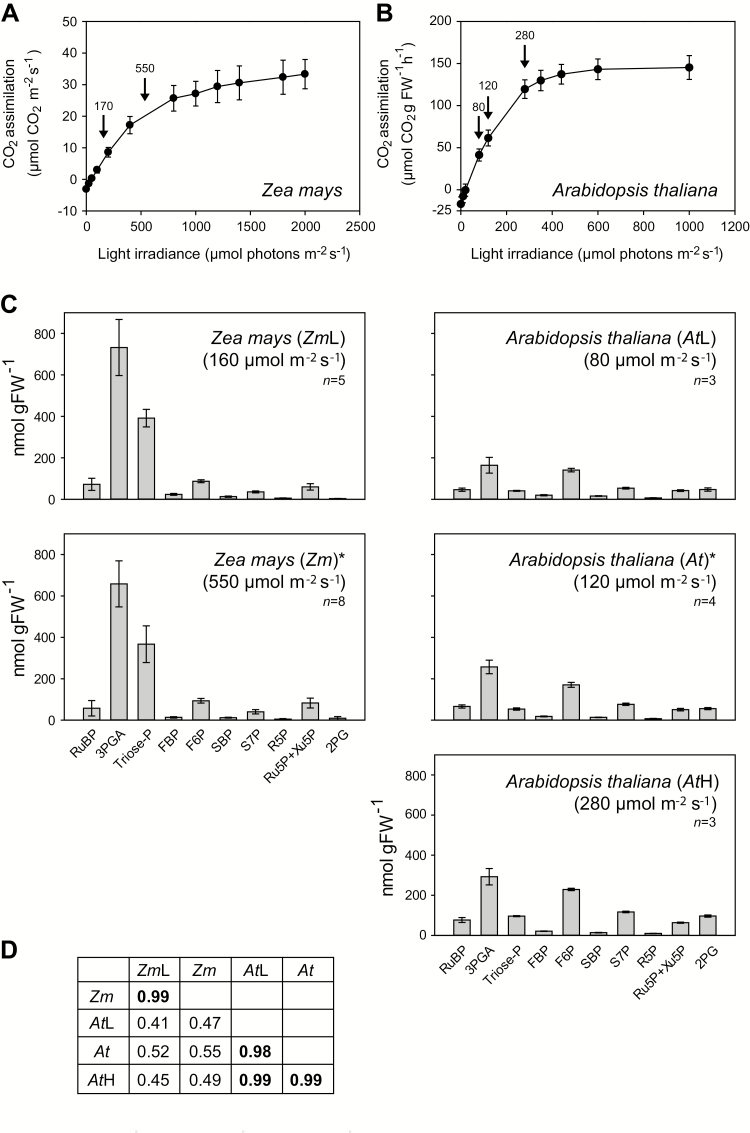
CO_2_ assimilation rate in *Z. mays* and *A. thaliana*, and CBC metabolite profiles in *Z. mays* and *A. thaliana* at different short-term irradiances. *Zea mays* and *A. thaliana* were grown at 550 µmol m^−2^ s^−1^ and 120 µmol m^−2^ s^−1^ irradiance, respectively. CO_2_ assimilation rate in (A) *Z. mays* (*n*=10) and (B) *A. thaliana* (*n*=9). The results are shown as mean (µmol CO_2_ m^−2^ s^−1^ and µmol CO_2_ g FW^−1^ h^−1^, for *Z. mays* and *A. thaliana*, respectively) ±SD. Arrows indicate the irradiances at which leaves were sampled for metabolite analysis. (C) *Zea mays* was harvested at growth irradiance (*Zm*, medium irradiance) or after being subjected for 4 h to 160 µmol m^−2^ s^−1^ (*Zm*L, low irradiance) from the beginning of the light period. *Arabidopsis thaliana* was harvested at growth irradiance (*At*, medium irradiance) or subjected for 15 min to 80 µmol m^−2^ s^−1^ or 280 µmol m^−2^ s^−1^ (*At*L, low and *At*H, high irradiances, respectively). Quenching of metabolism and harvest of leaf tissue were performed at least 4 h after the beginning of the light period. 2PG amounts are multiplied by 10 for better visibility. Asterisks indicate graphs already presented in Fig. 1. The results are shown as mean (nmol g FW^−1^) ±SD. (D) Correlation analysis. The metabolite data shown in (B) and (C) were used to perform Pearson’s correlation analysis between data sets from the same species at different irradiances, and correlations between different species. Before performing the correlation analysis, each data set was normalized by calculating the amount of carbon in a given metabolite, and dividing it by the total carbon in all metabolites in that data set. This was done to avoid secondary correlation due to any interspecies differences in leaf composition. The results are given as *r* and the higher correlations are indicated in bold. All correlations were positive. The original data are presented in Supplementary Dataset S1.

One potential complication of a cross-species comparison is that each species has a different light saturation response, making it difficult to standardize growth and harvest conditions across species. We grew and harvested all species at moderate and limiting irradiance, using lower irradiance for species whose photosynthesis saturates at lower light intensities (Supplementary Table S1). In addition, for *Z. mays* and *A. thaliana*, we asked whether short-term changes in irradiance lead to major changes in the metabolite profile, using an additional lower irradiance for *Z. mays* (Fig. 2A, covering the range from 40% to 133% of that required for half-maximal rates of photosynthesis), and a lower and a higher near-saturating irradiance for *A. thaliana* (Fig. 2B, covering the range from 67% to 233% of that required for half-maximal rates of photosynthesis). The metabolite profiles were not greatly altered for either species (Fig. 2C), except that higher irradiance tended to lead to a general increase in metabolite levels. Metabolite levels in a given species were strongly correlated irrespective of irradiance (*r*>0.98), whereas metabolite levels were poorly correlated between species (Fig. 2D).

### Participation of pools in photosynthesis

Our approach assumes that the investigated metabolites are predominantly involved in the CBC. If they are also involved in another pathway, the total content will not provide reliable information about the size of the CBC pool. Published ^13^C labelling kinetics validate this assumption for *N. tabacum*, *A. thaliana*, and *Z. mays* (Hasunuma *et al.*, 2010; Szecowka *et al.*, 2013; Arrivault *et al.*, 2017); after pulsing with ^13^CO_2_, all of the CBC metabolites showed a rapid rise in ^13^C enrichment to reach a final value of ≥80%. One exception was SBP in maize, where ^13^C enrichment plateaued at ~14%. We performed analogous ^13^CO_2_ labelling experiments for *M. esculenta* which, like *Z. mays*, is a subtropical species adapted to high-light conditions. We also chose *M. esculenta* because it has been suggested to be a C_4_ or C_3_–C_4_ intermediate species (Cock *et al.*, 1987; El-Sharkawy and Cock, 1987). A subsequent study showed that *M. esculenta* performs C_3_ photosynthesis (Edwards *et al.*, 1990; see also De Souza *et al.*, 2017; De Souza and Long, 2018). Time-resolved ^13^CO_2_ labelling would provide a further test that *M. esculenta* is a C_3_ species

In *M. esculenta*, CBC intermediates rose rapidly to high (>75%) ^13^C enrichment (Supplementary Fig. S3A; Supplementary Dataset S2) except for SBP where enrichment plateaued at ~40% and about half of the SBP remained in the unlabelled form after 60 min (Supplementary Fig. S3B). Otherwise, the labelling time series in *M. esculenta* resembled published time series for the C_3_ plants *A. thaliana* (Szecowka *et al.*, 2013) and *N. tabacum* (Hasunuma *et al.*, 2010). In particular, labelling of 4-C acids was very slow (Supplementary Fig. S3C).

### Chlorophyll and protein

**Fig. 3.**
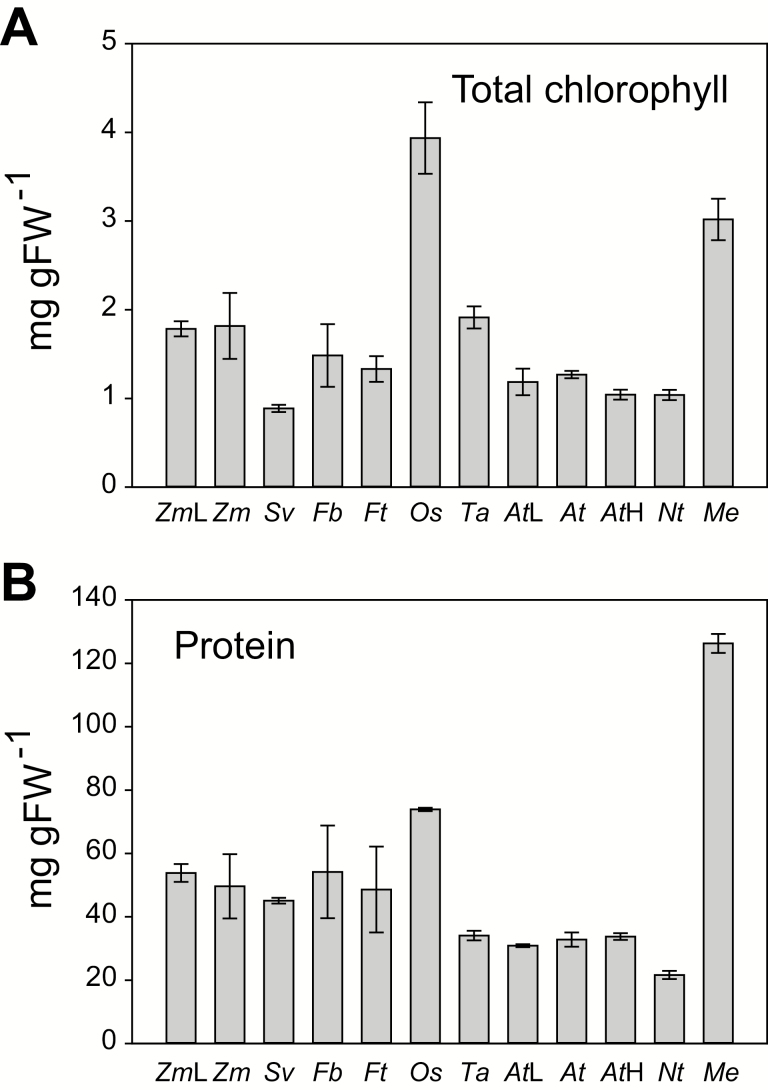
Total chlorophyll (A) and protein (B) content in different species. Measurements were performed in *Z. mays* at low and medium irradiance (*Zm*L and *Zm*, respectively), *S. viridis* (*Sv*), *F. bidentis* (*Fb*), *F. trinervia* (*Ft*), *O. sativa* (*Os*), *T. aestivum* (*Ta*), and *A. thaliana* at low, medium, and high irradiance (*At*L, *At*, and *At*H, respectively), *N. tabacum* (*Nt*) and *M. esculenta* (*Me*). Growth and harvest conditions can be found in Supplementary Table S1. The results are shown as mean (mg g FW^−1^) ±SD. The original data are presented in Supplementary Dataset S1.

Leaf composition varies between species (see the Introduction). This could contribute to interspecific differences in absolute metabolite levels; in particular, differences in leaf composition could lead to systematically higher or lower levels of all metabolites. We therefore determined total chlorophyll and protein contents in the leaf material used for metabolite analyses. Total chlorophyll content (Fig. 3A) was similar on a FW basis in all species except for *O. sativa* and *M. esculenta*, which had considerably higher values. Protein content on a FW basis (Fig. 3B) was similar in all species except for lower values in *N. tabacum*, and higher values in *O. sativa* and, especially, *M. esculenta.* These results partly explain why CBC metabolite levels on a FW basis tended to be low in *N. tabacum* and high in *O. sativa* and *M. esculenta* (Fig. 1).

### Principal component analysis

**Fig. 4.**
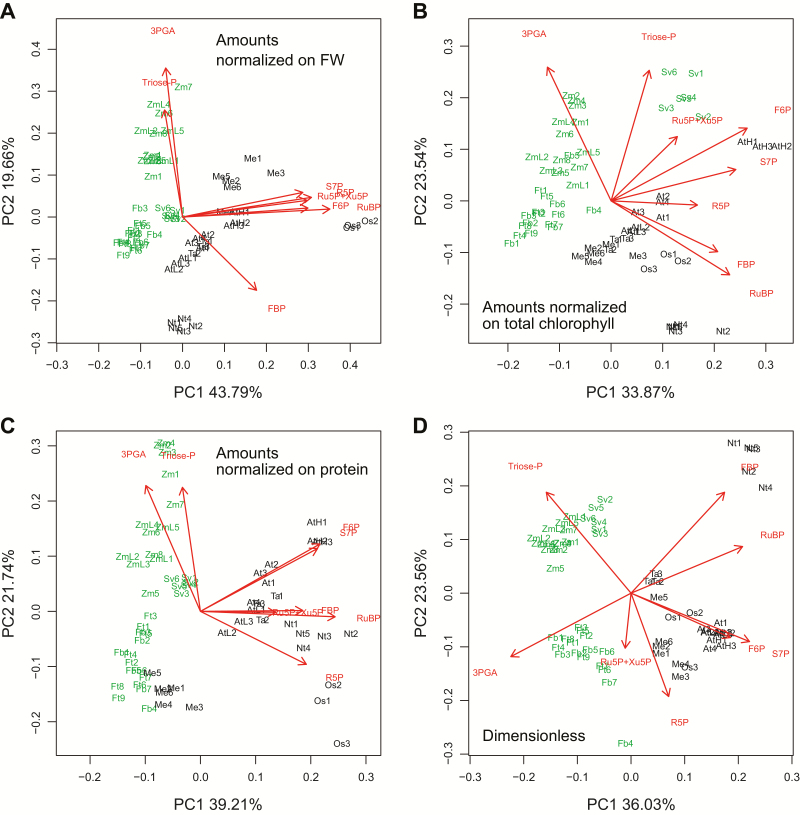
Principal component analyses of the CBC metabolite profiles in all tested species. The analyses were performed on the metabolite data set excluding 2PG and SBP (2PG was omitted to avoid systematic bias between C_3_ and C_4_ species due the differing rates of RuBP oxygenation, and SBP was omitted because in some species part of the pool may not be involved in the CBC; see text for details). Metabolite amounts were normalized on (A) FW, (B) total chlorophyll content, or (C) protein content, or (D) were transformed into a dimensionless data set. For dimensionless data set determination, in a given sample, the level of each metabolite was first transformed to C equivalent values by multiplying the amount (nmol g FW^−1^) by the number of C atoms in the metabolite. The C equivalent amounts of all CBC intermediates plus 2PG were then summed. In the last step, the C equivalent value of a given metabolite was divided by the summed C equivalent values. The transformed values and calculation steps are provided in Supplementary Dataset 1. This transformation generates a dimensionless data set (provided in Supplementary Dataset S1) in which each metabolite receives a value equal to its fractional contribution to all the C in CBC metabolites plus 2PG. As this data set is dimensionless, there is no systematic bias due to differences in leaf composition. The distribution of C_4_ species (green) and C_3_ species (black) is shown on PC1 and PC2 (*Z. mays*, Zm and ZmL; *S. viridis*, Sv; *F. bidentis*, Fb; *F. trinervia*, Ft; *O. sativa*, Os; *T. aestivum*, Ta; *A. thaliana*, AtL, At, and AtH; *N. tabacum*, Nt; *M. esculenta*, Me). The loadings of CBC intermediates in PC1 and PC2 are shown in red. Principal component analyses with the full metabolite data set and with all metabolites except either 2PG or SBP are shown in Supplementary Fig. S4 (amounts normalized on FW), Supplementary Fig. S5 (amounts normalized on total chlorophyll content), Supplementary Fig. S6 (amounts normalized on protein content), and Supplementary Fig. S7 (dimensionless). The original data are presented in Supplementary Dataset S1.

We performed principal component (PC) analyses to provide an integrated overview of the CBC metabolite profiles in the nine species. PC analysis gives information about which samples (here, different species) are closely related or separated, and which variables (here, metabolites) contribute to this relationship. The analysis was performed with *z*-scored data (i.e. normalizing the individual values of a given variable on the mean value for that variable) to ensure that each metabolite adopted an equally important role in the analysis, independent of its absolute abundance. Each individual sample was included separately in the analysis to provide an overview of the quality of within-species replication. We included the low light maize and the low and high light Arabidopsis samples to further test the impact of prevailing irradiance. In the analyses shown in Fig. 4, we omitted 2PG to focus solely on the CBC and exclude effects due to lower photorespiration in C_4_ plants. We also omitted SBP because of the labelling data (Supplementary Fig. S3; Arrivault *et al.*, 2017) indicating that in some species part of the SBP pool is not involved in the CBC. For comparison, analyses including 2PG and SBP are provided in Supplementary Figs S4–S7.

As previously mentioned, some cross-species variation in metabolite levels may be driven by changes in leaf composition. We therefore performed PC analyses on data sets in which the metabolites were normalized on FW (Fig. 4A; Supplementary Fig. S4), total chlorophyll content (Fig. 4B; Supplementary Fig. S5), or protein content (Fig. 4C; Supplementary Fig. S6). We also performed PC analysis on a dimensionless data set in which, for a given species, the amount of C in a given metabolite was divided by the total amount of C in all CBC intermediates plus 2PG (Fig. 4D; Supplementary Fig. S7). In total, we performed 16 PC analyses with different metabolite data sets and normalizations. In interpreting the plots, we focused on features that were seen in all or the vast majority of these analyses.

In analyses with the FW-, chlorophyll-, and protein-normalized data sets and the dimensionless data set, PC1 accounted for 44–46, 33–35, 34–36, and 39–40%, respectively, of the total variance, while PC2 accounted for 17–20, 20–23, 21–22, and 19–22%, respectively (Fig. 4A–C; Supplementary Figs S4–S6). In all cases, replicates for a given species grouped together, showing that within-species variance was smaller than interspecies differences. This included samples harvested at low and ambient light intensities for *Z. mays* and for *A. thaliana*. The *A. thaliana* samples collected at high light grouped separately from the other *A. thaliana* samples, but well removed from the other species in PC analyses with the FW-, chlorophyll-, and protein-normalized data sets. In PC analyses with the dimensionless data set, *A. thaliana* samples from all three light intensities grouped together (Fig. 4D; Supplementary Fig. S7), showing that increasing light intensity led mainly to a general increase in metabolite levels rather than to changes in their relative levels.

Inspection of the species distribution in the PC plots leads to three main conclusions. First, the PC analyses almost always separated C_4_ species from C_3_ species; this holds irrespective of how the metabolite data are normalized, and whether 2PG and SBP were excluded (Fig. 4) or included (Supplementary Figs S4–S7). *Manihot esculenta* showed a slight overlap with the *Flaveria* spp. in the analyses using metabolites minus SBP and 2PG, when the data set was normalized on protein (Fig. 4C), but was fully separated from all of the C_4_ species in the 15 other PC analyses. *A. thaliana* in low light showed a slight overlap with *Z. mays* or *S. viridis* in two (all metabolites normalized on FW, metabolites minus SBP normalized on FW; Supplementary Fig. S4) of the 16 data permutations. Secondly, within the C_4_ species, *Z. mays* and *S. viridis* separated from each other and from the *Flaveria* spp. in most of the PC analyses, while the two *Flaveria* spp. always overlapped with each other. Thirdly, the five C_3_ species were almost always clearly separated from each other. In the analyses based on FW-normalized data, *O. sativa* and *M. esculenta* separated strongly from other C_3_ species in PC1 (Fig. 4A; Supplementary Fig. S4). This was less marked in the PC analysis based on chlorophyll- or protein-normalized data (Fig. 4B, C; Supplementary Figs S5, S6), indicating that the strong separation in the analysis with FW-normalized data is partly driven by secondary effects due to leaf composition. Similarly, *N. tabacum* was less strongly separated from the other four C_3_ species in the PC analysis with protein-normalized data than with FW- or chlorophyll-normalized data. Despite these small shifts in the relationships, the five C_3_ species still separated from each other in the PC analyses with the chlorophyll- and protein-normalized data sets, as well as with the dimensionless data set (Fig. 4D; Supplementary Fig. S7).

The metabolite loadings (Fig. 4; Supplementary Figs S4–S7) reveal that the separation of C_4_ from C_3_ species was driven not only by lower levels of RuBP and (when included) 2PG, but also by other CBC metabolites. 3PGA and triose-P contributed to the separation of the C_4_ species *Z. mays* and *S. viridis* from *F. trinervia* and *F. bidentis* (see the Discussion). Almost every metabolite contributed to the separation between the five C_3_ species, with large contributions from RuBP, FBP, F6P, S7P, ribose 5-phosphate (R5P), triose-P, and 3PGA.

**Fig. 5.**
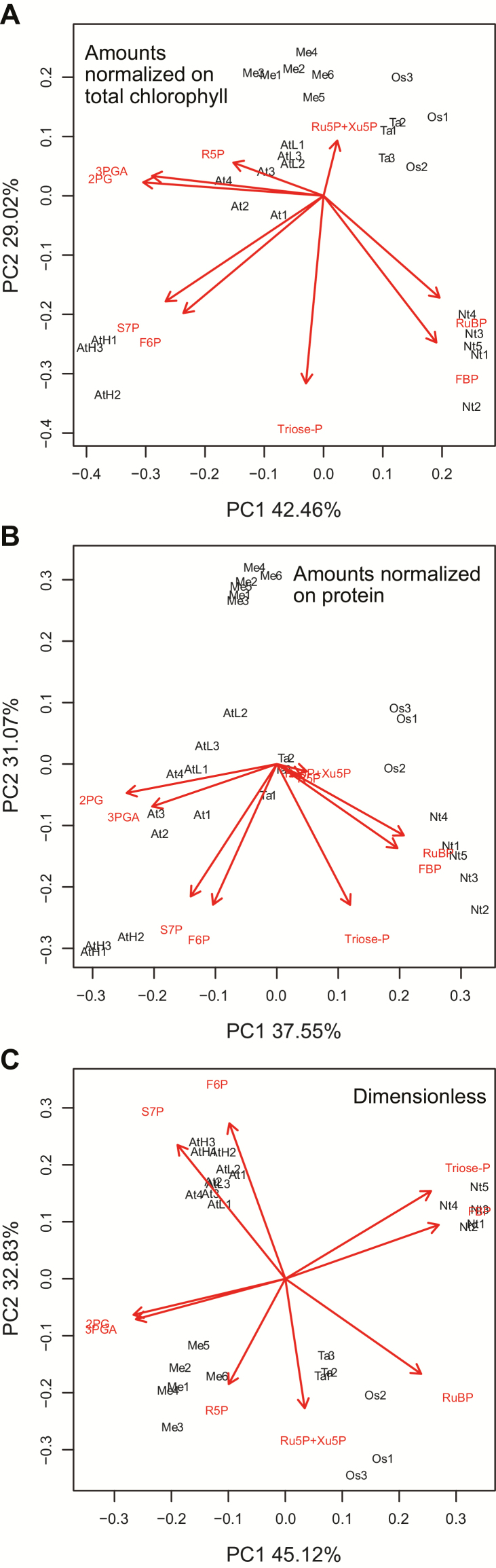
Principal component analysis of the CBC metabolite contents in C_3_ species only. The analyses were performed on the metabolite data set excluding SBP. Metabolite data were normalized on (A) total chlorophyll content and (B) total protein, and (C) using a dimensionless data set (see legend of Fig. 4). The distribution of C_3_ species is shown on PC1 and PC2 (*O. sativa*, Os; *T. aestivum*, Ta; *A. thaliana*, AtL, At, and AtH; *N. tabacum*, Nt; *M. esculenta*, Me). The loadings of CBC intermediates in PC1 and PC2 are shown in red. PC analyses with the full metabolite data set and with all metabolites except either 2PG or SBP are shown in Supplementary Fig. S8 (amounts normalized on total chlorophyll content), Supplementary Fig. S9 (amounts normalized on total protein), and Supplementary Fig. S10 (dimensionless). The original data are presented in Supplementary Dataset S1.

We repeated the PC analysis on a data set including only C_3_ species and with metabolites normalized on total chlorophyll content or protein content, and with a dimensionless data set (Fig. 5; Supplementary Figs S8–S10). Replicate samples from a given species grouped closely together. *A. thaliana*, *N. tabacum*, and *M. esculenta* were clearly separated from *T. aestivum* and *O. sativa*, which were only weakly separated. The high irradiance *A. thaliana* samples grouped separately from the low and medium light *A. thaliana* samples, but in the same tangent, and were clearly separated from the other four C_3_ species. Metabolite loadings revealed strong contributions from 3PGA, triose-P, RuBP, FBP, F6P, and S7P to the separation.

### Coefficient of variance

**Fig. 6.**
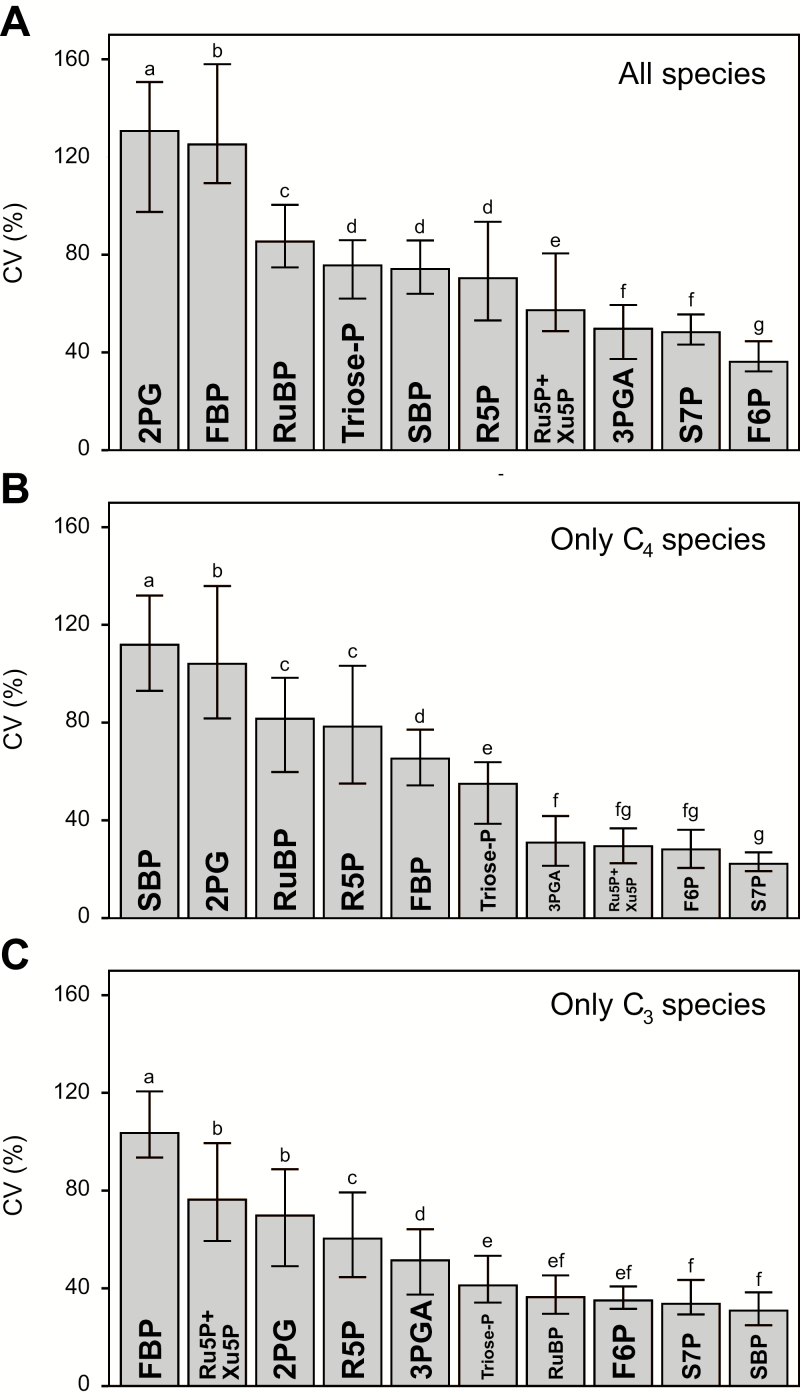
Coefficient of variance (CV) of CBC metabolites between species. The CV (SD/mean×100) is a standardized quantity describing the dispersion of a population distribution (Simpson and Roe, 1939). The analysis was performed on a dimensionless data set that was generated as described for Fig. 4D. The transformed data (presented in Supplementary Dataset S1) were used to calculate the bootstrapped CV for each metabolite (30 bootstrap iterations). The 95% confidence interval was estimated using the basic bootstrap method. Statistically significant differences between metabolites are indicated by letters (ANOVA on the bootstrap results followed by the Tukey’s HSD post-test). (A) All species, (B) only C_4_ species, and (C) only C_3_ species.

We calculated the coefficient of variance (CV) to determine which metabolites showed the greatest interspecies variance for all nine species together, for the four C_4_ species, and for the five C_3_ species (Fig. 6). To avoid influence due to leaf composition, this analysis was performed on the dimensionless data set. Across all species (Fig. 6A), the highest CV was for 2PG, followed by FBP, RuBP, triose-P, SBP, and R5P. When only C_4_ species are considered (Fig. 6B), the highest CV was for SBP, followed by 2PG, RuBP, R5P, FBP, and triose-P. When only C_3_ species are considered (Fig. 6C), the highest CV was for FBP, followed by Ru5P+Xu5P, 2PG, R5P, and 3PGA.

### Correlation analysis

**Fig. 7.**
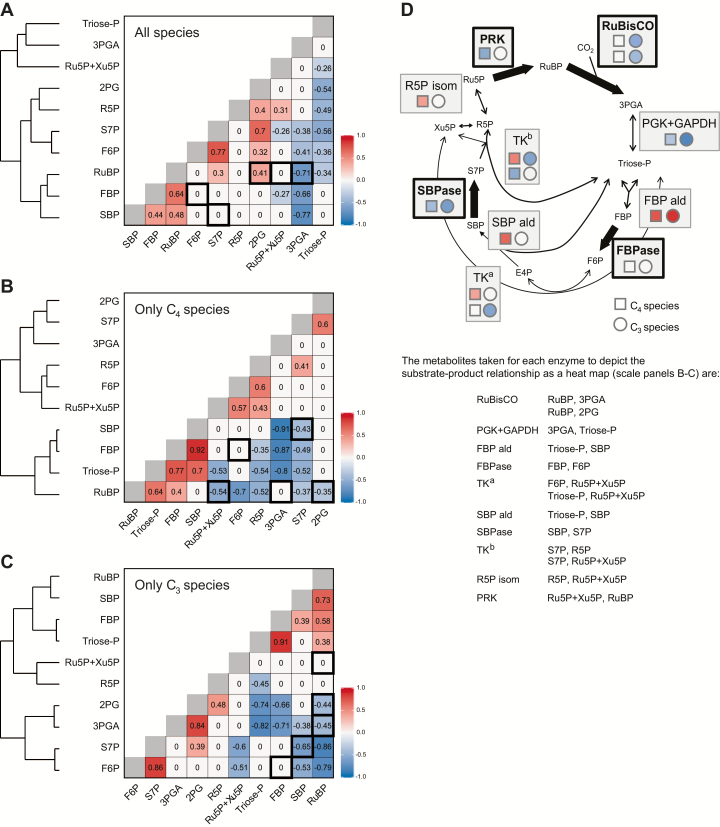
Correlation between levels of CBC metabolites. The correlations were performed with all individual samples from a dimensionless data set, generated as described for Fig. 4D. The transformed data were used to calculate the Pearson’s correlation matrix on every pair of metabolites. Correlation values are given in the figure panels and indicated by a heat map. The adjacent dendrograms show clusters defined using the complete linkage method (Sørensen, 1948). Non-significant correlations (*P*≥0.05; two-sided Student’s *t*-test) are set as zero. Metabolite pairs that are linked by irreversible reactions are indicated by a black box. (A) All species, (B) only C_4_ species, and (C) only C_3_ species. An alternative display is provided in Supplementary Fig. S11, with the same fixed order of metabolites in each panel, corresponding to the reaction sequence in the CBC. The same heat map scale is used for (A–C). (D) Schematic representation of interspecies variance in the ratio of substrate abundance:product abundance for different CBC enzymes. Enzymes that catalyse irreversible reactions are highlighted in bold. For each enzyme reaction, the substrate and product that were compared are indicated in the list below the display. This display is schematic because some metabolites were not measured (erythrose 4-phosphate, E4P; and glyceraldehyde 3-phosphate, GAP) or were not separated (Ru5P and Xu5P). For reactions using GAP, it is assumed that GAP and dihydroxyacetone phosphate (DHAP) are in equilibrium. For transketolase (TK), two reactions were separated (termed TK^a^ and TK^b^). For TK^a^, the reactant E4P was missing, and only the relationships between triose-P and F6P and Ru5P+Xu5P are shown. For Tk^b^, the plot focuses on the relationship between S7P and R5P or Ru5P+Xu5P. The display shows the alternative pairs of metabolites compared, with the upper and lower symbols in the display corresponding to the upper and lower pair in the list. A similar display mode is used for the carboxylation and oxygenation reactions of Rubisco. The correlation coefficients are taken from (B) and (C), using the same heat map scale. Results are shown separated for correlations between the four C_4_ species (squares) and the five C_3_ species (circles). The analysis is not shown for the combined C_4_ plus C_3_ species set because, in this case, some relationships are driven by differences between C_4_ and C_3_ species. Additional abbreviations: fructose 1,6-bisphosphate aldolase (FBP ald), phosphoglycerate kinase (PGK), ribose 5-phosphate isomerase (R5P isom), sedoheptulose 1,7-bisphosphate aldolase (SBP ald).

When metabolite profiles are compared across different genotypes, they typically generate a correlation network (Meyer *et al.*, 2007; Sulpice *et al.*, 2009, 2013; Zhang *et al.*, 2015; Wu *et al.*, 2016). This reflects features of the underlying metabolic pathways that are maintained across genotypes and generate conserved relationships between metabolites. Our data set allowed us to apply this approach to interspecies variation in the CBC. We performed pairwise PC analysis and clustering on CBC metabolites (Fig. 7) using the dimensionless data set to avoid bias from changes in leaf composition. We also searched for relationships between 2PG and the CBC metabolites. Metabolite pairs that are linked by irreversible reactions (Bassham and Krause, 1969; Mettler *et al.*, 2014) are indicated by black boxes in the figure. Correlation analysis and clustering were performed for all nine species (Fig. 7A), for the four C_4_ species (Fig. 7B), and for the five C_3_ species (Fig. 7C). To aid visual comparison across the species sets, correlation coefficients are also shown in Supplementary Fig. S11 with the metabolites in a fixed order corresponding to CBC topology.

The CBC correlation network for all nine species (Fig. 7A) contained six positive correlations (e.g. F6P versus S7P; all pairwise comparisons between RuBP, FBP, and SBP), many non-significant relationships [e.g. FBP versus F6P; SBP versus S7P; RuBP versus R5P and Ru5P+Xu5P (here collectively called pentose-P)], and 13 negative correlations (e.g. 3PGA or triose-P versus most other CBC metabolites). In some cases, the correlations were driven by differences between C_4_ and C_3_ species; for example, the positive correlation between 2PG and RuBP is driven by the lower levels of both metabolites in C_4_ compared with C_3_ species (see Fig. 1). However, in many cases, the correlations were also seen within the subset of C_4_ and within the subset of C_3_ species (see Supplementary Fig. S11 and below).

The correlation network for CBC metabolites in C_4_ species (Fig. 7B) contained nine positive (e.g. FBP versus SBP; FBP versus RuBP; and triose-P versus FBP, SBP, and RuBP) and 13 negative (e.g. 3PGA versus triose-P, FBP, and SBP; triose-P versus S7P and pentose-P; RuBP versus F6P, S7P, and pentose-P; and SBP versus S7P) relationships. There was no significant relationship between FBP and F6P. The correlation network for CBC metabolites in C_3_ species (Fig. 7C) contained six positive (e.g. all pairwise comparisons between RuBP, FBP, and SBP; and F6P versus S7P) and 11 negative (e.g. 3PGA versus triose-P, FBP, SBP, and RuBP; RuBP versus F6P and S7P; and SBP versus S7P) relationships. There was no significant relationship between FBP and F6P, or between RuBP and pentose-P. 2PG correlated positively with S7P and negatively with RuBP in C_4_ and C_3_ species, respectively, and positively with 3PGA and negatively with triose-P and FBP in C_3_ species.

The correlation networks can be interpreted by relating them to CBC topology (Fig. 7D; see also Supplementary Fig. S11). Figure 7D focuses on correlations seen within the subset of C_4_ species and within the subset of C_3_ species. Triose-Ps are used to synthesize FBP and SBP in reversible reactions catalysed by aldolase. This may explain the positive correlations between triose-P and FBP or SBP (except for SBP in C_3_ plants). FBPase and SBPase catalyse irreversible reactions. The non-significant or negative correlations between FBP and F6P and between SBP and S7P point to interspecies variance in the regulation of FBPase and SBPase. This may also explain the absence of a positive correlation between triose-P and pentose-P that otherwise might have been expected because pentose-Ps are formed from triose-P and F6P or S7P in reversible reactions catalysed by transketolase (TK). The negative relationship between pentose-P and RuBP points to interspecies variation in the regulation of PRK. Further, the positive correlations of FBP and SBP with RuBP (see Supplementary Fig. S11) indicate that FBPase and SBPase activity vary reciprocally to PRK activity and/or co-ordinately with binding or use of RuBP by RuBisCO.

## Discussion

The CBC is an ancient pathway that has been under selective pressure due to the long-term increase of the O_2_:CO_2_ ratio in the atmosphere and particularly over the last 30 million years due to falling CO_2_ concentrations, which led to independent evolution of a CCM in >100 terrestrial plant lineages. However, the vast majority of terrestrial species did not evolve a CCM, probably because they were unable to follow the multistep evolutionary trajectory that was required to acquire this complex trait (Sage *et al.*, 2012; Christin and Osborne, 2013; Heckmann *et al.*, 2013). Present-day C_3_ plants nevertheless will have been subject to similar selective pressures to those that drove the evolution of C_4_ or CAM photosynthesis. Indeed, in the absence of a CCM, the selective pressures on the CBC may have been even greater than in plants that did evolve a CCM. In addition to low CO_2_, it is likely that environmental factors such as irradiance, temperature, and nutrient and water availability exerted more or less selective pressure, depending on the local environment, and leading to different evolutionary trajectories in different populations. While it is well documented that there is large variation in photosynthetic rate between terrestrial species (Evans, 1989; Wullschleger, 1993; Lawson *et al.*, 2012), previous studies of the underlying causes have focused on leaf morphology and composition (Field and Mooney, 1986; Evans, 1989; Hikosaka, 2010; Poorter *et al.*, 2015; Díaz *et al.*, 2016), stomatal conductance (Lawson *et al.*, 2012), and the kinetic characteristics of RuBisCO (Yeoh *et al.*, 1980; Jordan and Ogren, 1981; Badger *et al.*, 1998; Tcherkez *et al.*, 2006; Galmés *et al*., 2014*b*; Prins *et al.*, 2016; Sharwood *et al.*, 2016a, b). Little is known about whether the CBC operates in a highly conserved manner or in different modes in different C_3_ species.

We have used metabolite profiling as an unbiased strategy to search for interspecific variance in CBC operation. The underlying assumption is that changes in the balance between different enzymatic steps will lead to changes in the relative levels of pathway intermediates. This approach is top down, in the sense that it does not make assumptions about whether the observed variance is due to changes in gene expression and protein abundance, enzyme kinetics, or regulatory networks that act on the enzymes. We applied it to search for differences in CBC operation between C_4_ and C_3_ plants, and within C_3_ species. As our aim was to compare CBC operation across species, we focused exclusively on the metabolites that are involved in the CBC plus 2PG, the immediate product of the RuBisCO oxygenation reaction. We excluded metabolites involved further downstream in photorespiration and metabolites involved in the CO_2_-concentrating shuttle in C_4_ plants, which have non-photosynthetic functions in C_3_ plants.

Our interspecies comparison required important control experiments and cross-checks during data analysis. First, plant species differ in their photosynthetic rate and its dependence on light, temperature, and the availability of water, nutrients, and CO_2_ (see the Introduction). We grew and harvested plants in a light regime that was limiting for that species, rather than using identical conditions for all species. In these conditions, RuBP regeneration is likely to be limiting, and effects of light stress are avoided. Importantly, we showed for one C_4_ species (*Z. mays*) and one C_3_ species (*A. thaliana*) that although increased harvest irradiance led to higher levels of metabolites, it did not strongly alter their relative levels (Fig. 2). Secondly, it is important that the CBC pool accounts for most or all of the total content of a given metabolite. Analysis of published data for two C_3_ (*N. tabacum* and *A. thaliana*), one C_4_ (*Z. mays*) species (Hasunuma *et al.*, 2010; Szecowka *et al.*, 2013; Arrivault *et al.*, 2017), and a new data set for the C_3_ species *M. esculenta* (Supplementary Fig. S3) showed that CBC intermediates exhibit a rapid rise in ^13^C enrichment to a high level after supplying ^13^CO_2_. This provides evidence that most of the total pool is indeed involved in the CBC. This conclusion is supported by published subcellular fractionation studies, in which most CBC intermediates are exclusively or largely confined to the plastid (Gerhardt *et al.*, 1987; Szecowka *et al.*, 2013). The only exception was SBP, which was only partially labelled in *Z. mays* and *M. esculenta*. We do not know whether there is a separate pool of SBP that is not involved in CO_2_ fixation, or if these plant species contain an unknown metabolite with an identical chromatographic behaviour, mass, and fragmentation pattern to SBP. In our interpretation of the metabolite profiles, we took care that our conclusions did not depend on inclusion of SBP. A third set of controls addressed the issue that leaf composition varies between species, with the result that absolute values for metabolite content will depend on the unit in which they are given. We analysed metabolite data normalized on FW, chlorophyll, or protein content, and also used a dimensionless data set in which metabolite levels were expressed relative to each other. Our interpretation focused on results that were independent of how the data were normalized. Importantly, inclusion of the dimensionless data set eliminated secondary correlations due to differences in leaf composition, and placed the emphasis on relative rather than absolute levels of metabolites. It minimizes contributions from differing light regimes, which had less effect on relative than on absolute metabolite levels (see above).

We included four C_4_ species in our panel to test if CBC profiles could distinguish between species in which it is known that the CBC operates in a different context from that of C_3_ plants. The CBC operates at a much higher intercellular CO_2_ concentration in C_4_ than in C_3_ plants, and RuBisCO has a higher affinity for CO_2_, and an increased catalytic rate in C_4_ compared with C_3_ species (see the Introduction). PC analysis confirmed that CBC metabolite profiles allow C_4_ and C_3_ species to be distinguished (Fig. 4; Supplementary Figs S4–S7). As expected, C_4_ species had lower 2PG and RuBP than C_3_ species (Fig. 1). However, the separation in the PC analysis was also seen when 2PG was excluded, and was driven by several other CBC intermediates, pointing to broader changes in CBC operation between C_4_ and C_3_ species.

The four C_4_ species belong to the NADP-malic enzyme C_4_ subtype. Interestingly, PC analysis separated Z. *mays* and *S. viridis* from the two *Flaveria* spp. Whilst this might reflect a difference between monocots and eudicots, the PC vectors indicated that this separation reflected higher levels of 3PGA and, in particular, triose-P in *Z. mays* and *S. viridis* (Fig. 4; Supplementary Figs S4–S7; see also Fig. 1). Most NADP-malic enzyme C_4_ subtypes, including *Z. mays*, have dimorphous chloroplasts with little or no PSII activity in the bundle sheath cells (Munekage, 2016). They operate an intercellular shuttle in which 3PGA moves from the bundle sheath to the mesophyll cells and is reduced to triose-P, which returns to the bundle sheath. Intercellular movement is thought to occur by diffusion (Hatch and Osmond, 1976), driven by concentration gradients that require the build-up of large pools of 3PGA and triose-P in the bundle sheath and mesophyll cells, respectively (Leegood, 1985; Stitt and Heldt, 1985; Arrivault *et al.*, 2017). *Flaveria bidentis* and *F. trinervia* can have PSII activity in the bundle sheath chloroplasts, although to a varying extent depending on conditions (Laetsch and Price, 1969; Höfer *et al.*, 1992; Meister *et al.*, 1996; Nakamura *et al.*, 2013). Their separation in the PC analysis from *Z. mays* and *S. viridis* might reflect decreased reliance on this intercellular shuttle.

Our panel included five C_3_ species, two monocots (*O. sativa* and *T. aestivum*) and three eudicots (*A. thaliana*, *N. tabacum*, and *M. esculenta*), with the individual species representing different phylogenetic lineages (Supplementary Fig. S1) and originating in differing climatic zones. The three C_3_ eudicot species represent two of the major lineages within the eudicots, namely the asterids (*N. tabacum*) and rosids (*A. thaliana* and *M. esculenta*), that contain 41% and 24% of all angiosperms, respectively. There was considerable interspecies variation in CBC metabolite profiles. This was evident from visual inspection of the metabolite levels (Fig. 1) and was confirmed by PC (Figs 4, 5; Supplementary Figs S4–S10) and variance (Fig. 6) analyses.

When metabolites were expressed on a FW basis, some of the variation was due to differences in leaf composition, with a strong trend to higher absolute levels in *O. sativa* and *M. esculenta*, reflecting their high chlorophyll and protein content. The high protein content in *O. sativa* may be linked to changes in leaf anatomy that enhance mesophyll transfer conductance, including small deeply lobed cells and densely arranged chloroplasts and stromules at the cell surface (Sage and Sage, 2009; Busch *et al.*, 2013). This high mesophyll transfer conductance may prevent internal CO_2_ from being drawn down by the high CBC activity that results from the high protein and metabolite content per unit FW in *O. sativa*. The high protein content in *M. esculenta* resembles the findings of previous reports (Awoyinka *et al.*, 1995; Nassar and Marques, 2006), and could explain the high rates of photosynthesis in this species.

However, the five C_3_ species still showed differing CBC metabolite profiles when metabolites were expressed on a chlorophyll or protein basis, or when the analyses were performed with a dimensionless data set. Variation was driven by many metabolites including RuBP, 3PGA, triose-P, FBP, F6P, S7P, and Ru5P+Xu5P. This variation points to different operating modes of the CBC in different C_3_ species. There were also differences in 2PG content; this might be related to the rate of RuBisCO oxygenation or removal of 2PG by 2-phosphoglycolate phosphatase.

Cross-species correlation analysis (Fig. 7; Supplementary Fig. S11) revealed that in both C_4_ and C_3_ species, the interspecies variance often included parallel changes of FBP, SBP, and RuBP, and unrelated or even reciprocal changes of these metabolites to F6P, S7P, and pentose-P. This is consistent with interspecies variation in the balance between FBPase, SBPase, PRK, and RuBisCO activity. It could reflect differences in the abundance or the regulation of these enzymes, both within C_3_ species and within C_4_ species, and between C_3_ and C_4_ species. Little is known about the expression, characteristics, and regulation of CBC enzymes in different species, with (see the Introduction) the exception of RuBisCO.

Our results do not reveal when and under what circumstances the variation in CBC function in C_3_ species appeared. It is tempting to link it with the selection pressure that led to the appearance of C_4_ and CAM photosynthesis, but it is likely to have started even earlier. Further, as pointed out by Zhu *et al.* (2007), it is possible that different C_3_ species are following different trajectories during the increase in CO_2_ levels in very recent evolutionary time. Our results also indicate that there is no strong connection between phylogeny and the diversity in CBC metabolite profiles in C_3_ species. In the PC analyses (Figs 4, 5; Supplementary Figs S4–S10), the two monocot species are often closely related, but the three eudicot species are highly diverse, and a given eudicot is often more closely related to the monocot species than to the other eudicot species. Unlike changes in genome sequence, complex emergent phenotypes may not accrue in a linear manner, and phylogenetically distinct species may undergo convergent evolution whilst phylogenetically related species may undergo divergent evolution, depending on the selective pressure they experience. Better understanding of the relationship between diversity in CBC profile, phylogeny, and evolution will require studies both with more phylogenetically diverse species and with more dense sampling in short evolutionary space.

In conclusion, marked differences in CBC metabolite profiles between five C_3_ species, including the major crop plants *O. sativum*, *T. aestivum*, and *M. esculenta*, and the important model plants *A. thaliana* and *N. tabacum*, reveal interspecies variation in the operating mode of the CBC in C_3_ plants. This probably reflects independent evolution of CBC regulation in different plant lineages, in analogy to the independent evolution of a CCM in different plant lineages. These findings, together with emerging evidence for interspecies variation in the properties of specific CBC enzymes (see the Introduction) and the growing realization that efficient photosynthesis requires integrated operation of the CBC (Stitt *et al.*, 2010; Raines, 2011; Simkin *et al.*, 2017), highlight the need for a mechanistic understanding of CBC regulation in a wider range of species. This will be an important step towards improving C_3_ photosynthesis and crop productivity.

## Supplementary data

Supplementary data are available at *JXB* online.

Fig. S1. Phylogenetic distribution based on APGIII of the tested plant species.

Fig. S2. Experimental set-up for ^13^CO_2_ labelling of *M. esculenta.*

Fig. S3. ^13^C enrichment (%) of CBC metabolites, relative abundance (%) of SBP isotopomers, and ^13^C enrichment (%) of malate, aspartate, pyruvate, and alanine in *M. esculenta.*

Fig. S4. PC analyses of all species using metabolite data normalized on FW (supplementary analyses to Fig. 4A).

Fig. S5. PC analyses on all species using metabolite data normalized on total chlorophyll content (supplementary analyses to Fig. 4B).

Fig. S6. PC analyses on all species using metabolite data normalized on protein content (supplementary analyses to Fig. 4C).

Fig. S7. PC analyses on all species using a dimensionless data set (supplementary analyses to Fig. 4D).

Fig. S8. PC analyses on C_3_ species only, using metabolite data normalized on total chlorophyll content (supplementary analyses to Fig. 5A).

Fig. S9. PC analyses on C_3_ species only, using metabolite data normalized on protein content (supplementary analyses to Fig. 5B).

Fig. S10. PC analyses on C_3_ species only, using a dimensionless data set (supplementary analyses to Fig. 5C).

Fig. S11. Correlation between levels of CBC metabolites, with metabolites shown in a fixed order reflecting the reaction sequence in the CBC.

Table S1. Growth conditions and photosynthetic rates.

Dataset S1. Metabolite levels, total chlorophyll, and protein contents in different species.

Dataset S2. Labelling kinetics of CBC and other intermediates after exposing *M. esculenta* to ^13^CO_2_ (supplementary data to Supplementary Fig. S3).

Supplementary Dataset S1

Supplementary Dataset S2

Supplementary Figure S1

Supplementary Table S1

## Abbreviations:

C: carbon
CAM: Crassulacean acid metabolism
CBC: Calvin–Benson cycle
CCM: carbon-concentrating mechanism
CV: coefficient of variance
DHAP: dihydroxyacetone phosphate
E4P: erythrose 4-phosphate
FBP: fructose 1,6-bisphosphate
FBPase: fructose-1,6-bisphosphatase
F6P: fructose 6-phosphate
GAP: glyceraldehyde 3-phosphate
NADP-GAPDH: NADP-glyceraldehyde-3-phosphate dehydrogenase
PC: principal component
PEP: phospho*enol*pyruvate
2PG: 2-phosphoglycolate
3PGA: 3-phosphoglycerate
PRK: phosphoribulokinase
R5P: ribose 5-phosphate
RuBP: ribulose 1,5-bisphosphate
Ru5P: ribulose 5-phosphate
SBP: sedoheptulose 1,7-bisphosphate
SBPase: sedoheptulose-1,7-bisphosphatase
S7P: sedoheptulose 7-phosphate
TK: transketolase
triose-P: triose phosphate
Xu5P: xylulose 5-phosphate
