# Supplementary material for: Metabolite profiles reveal interspecific variation in operation of the Calvin–Benson cycle in both C4 and C3 plants
Source: J Exp Bot. 2019 Feb 18;70(6):1843–58. doi: 10.1093/jxb/erz051 (PMC6436152; doi:10.1093/jxb/erz051)

Supplementary Figure S1. Phylogenetic distribution based on APGIII of the tested plant species. Adapted from Stevens, P. F. (2001 onwards). Angiosperm Phylogeny Website. Version 14, July 2017; http://www.mobot.org/MOBOT/research/APweb/

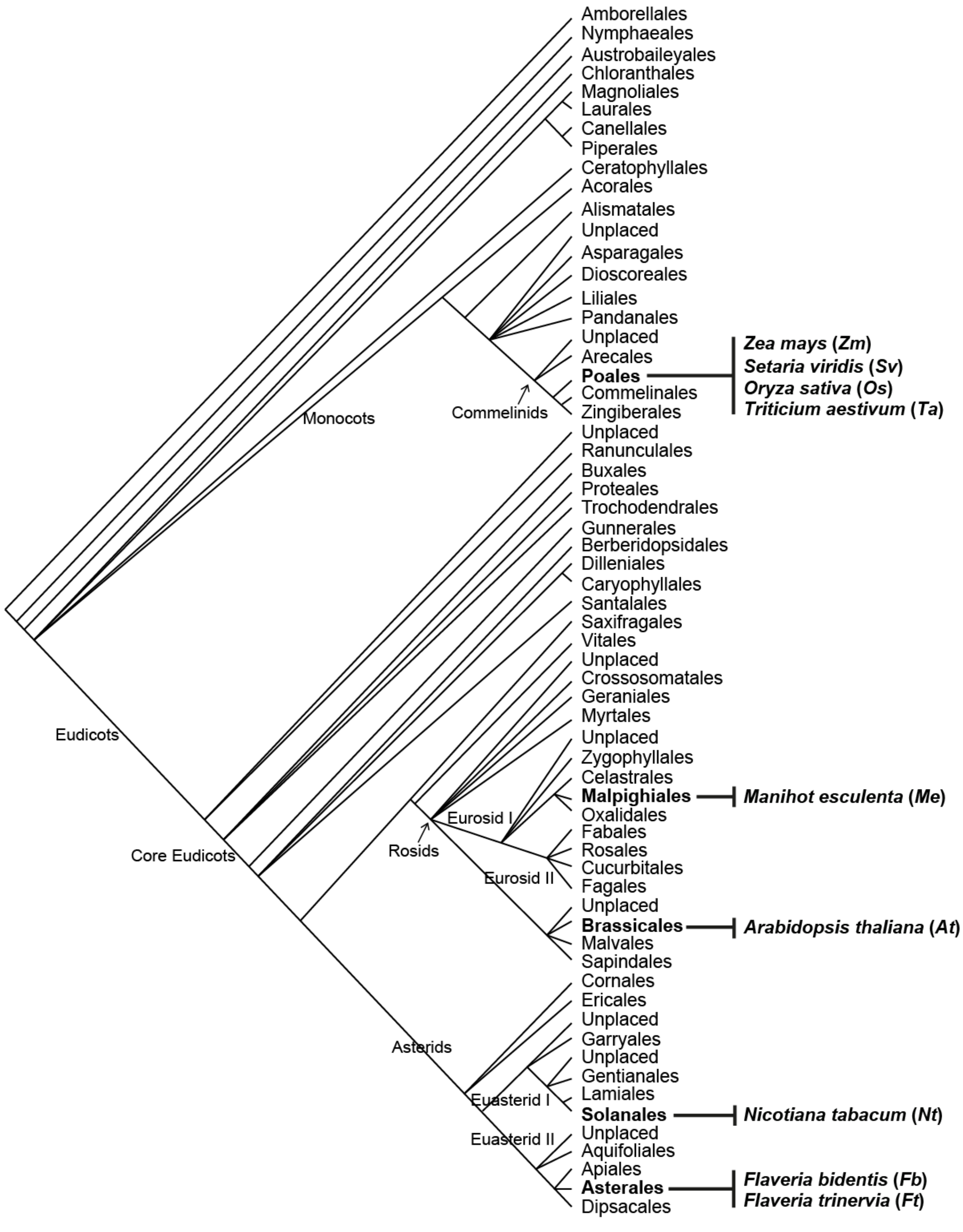

Figure S2. Experimental setup for $^{13}CO_2$ labelling of *M. esculenta.* (A) An example of a typical plant used for the feeding experiment. The fifth or sixth leaf from the top of the plant, indicated by the white arrow, was analyzed. **(B)** Overview of the experimental setup. The temperature in the labelling chamber was maintained by circulating water from a water bath. Additional light was supplied to the labelling chamber to compensate for the reduction of light intensity by the lid of the chamber. **(C)** Labelling chamber. The flow of the air is indicated by text. **(D)** Leaf used for the feeding experiment. A leaf disc was quenched from the centre leaf as indicated by the white circle.

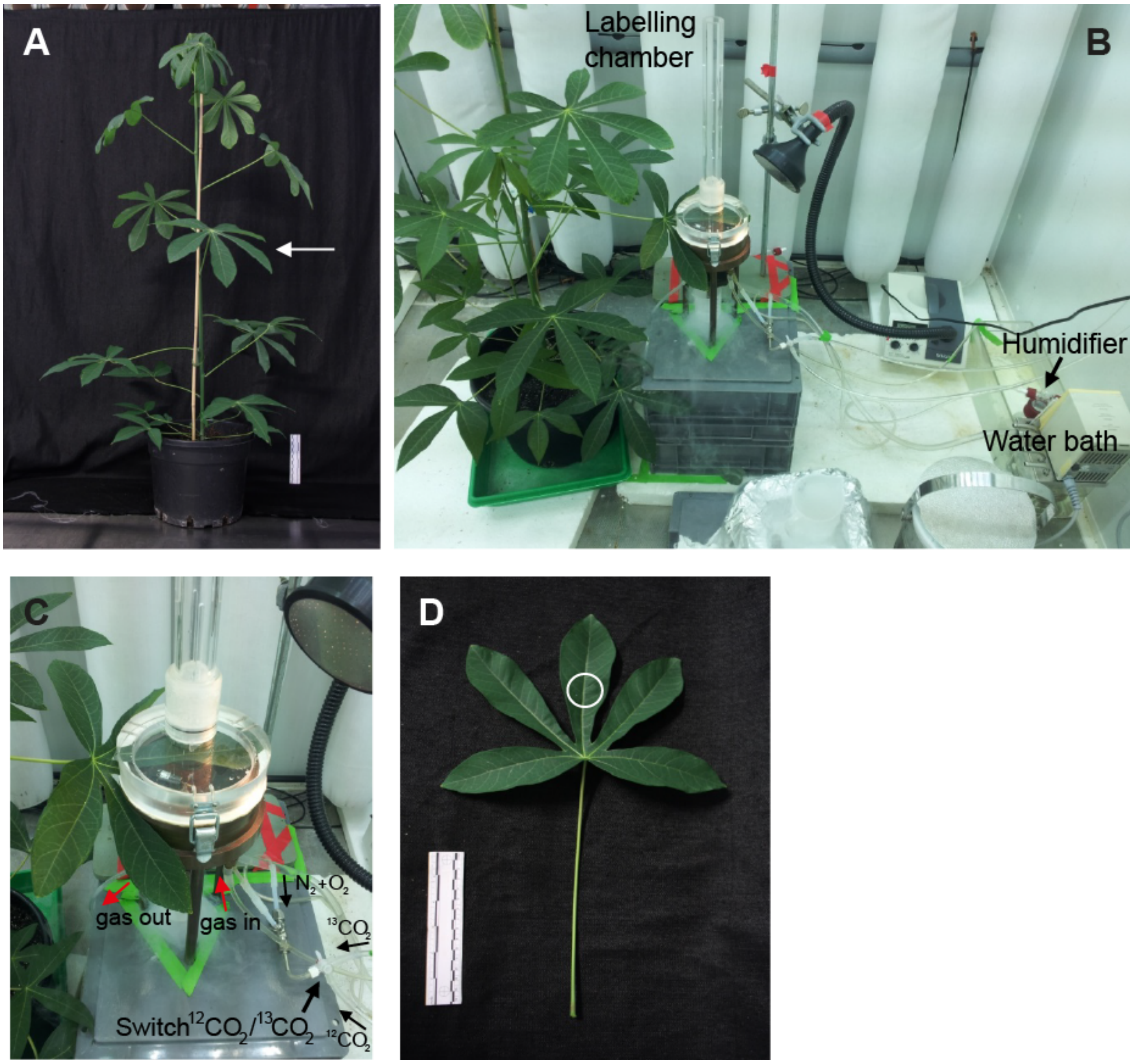

Supplementary Figure S3. **^{13}C enrichment (%) of CBC metabolites, relative abundance (%) of SBP isotopomers and ^{13}C enrichment (%) of malate, aspartate, pyruvate and alanine in *M. esculenta.* (A)** ^{13}C enrichment (%). **(B)** Relative abundance of each isotopomer (m_n) for SBP; n is the number of ^{13}C atoms incorporated. **(C)** ^{13}C enrichment (%) of malate, aspartate, pyruvate and alanine. When applicable, the results are shown as mean (n = 2 to 3) ± SD. The original data are presented in Supplementary Dataset S2.

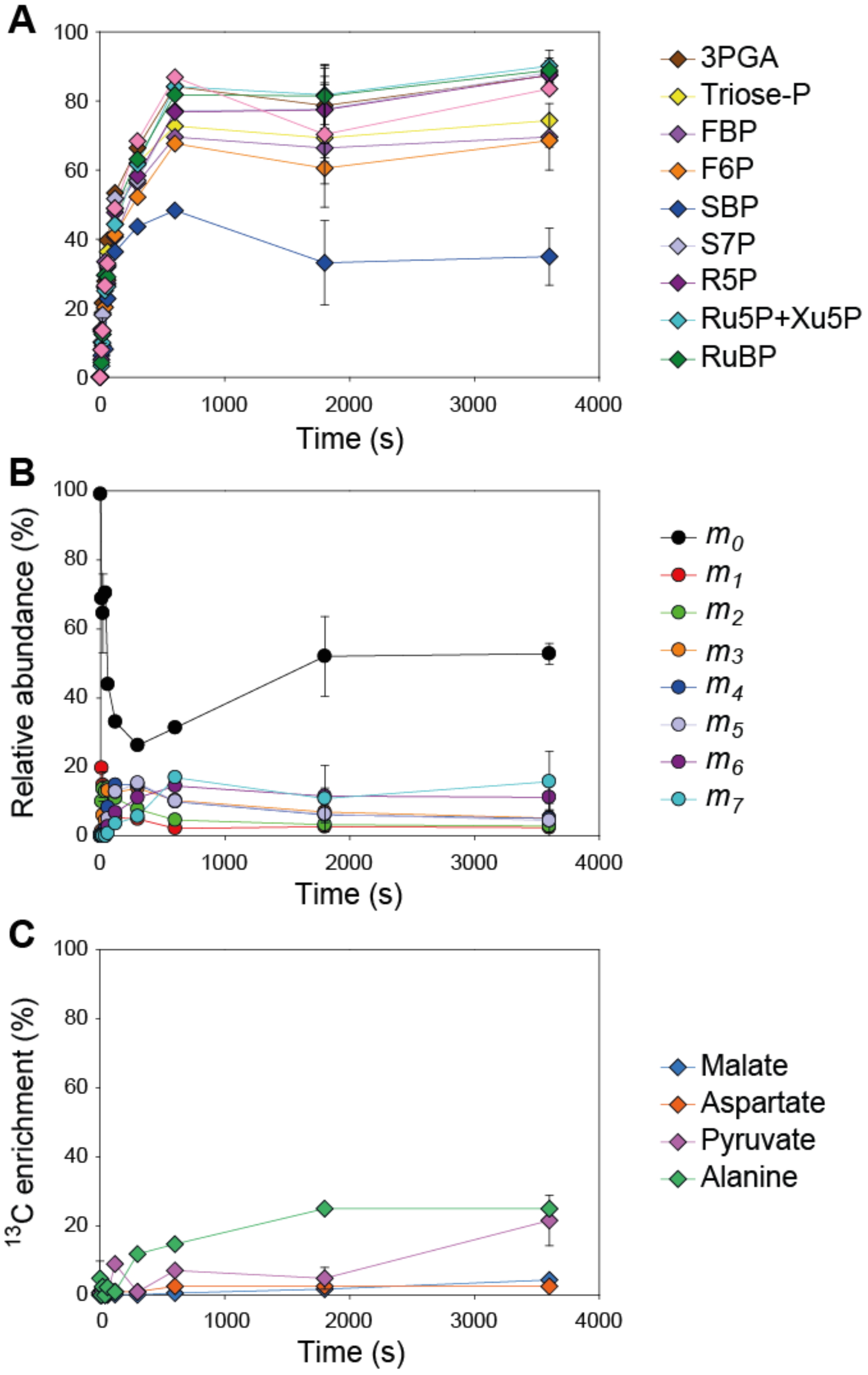

Supplementary Figure S4. PC analyses of all species using metabolite data normalized on FW (supplementary analyses to Figure 4A). Analyses were performed on the entire data set, or excluding 2PG, or excluding SBP, or excluding 2PG and SBP. The distribution of C_4 species (green) and C_3 species (black) is shown on PC1 and PC2 (*Z. mays,* Zm and ZmL*; S. viridis,* Sv; *F. bidentis,* Fb*; F. trinervia,* Ft*; O. sativa,* Os; *T. aestivum,* Ta*; A. thaliana,* AtL, At and AtH*; N. tabacum,* Nt*; M. esculenta,* Me). The loadings of CBC intermediates in PC1 and PC2 are shown in red. The original data are presented in Supplementary Dataset S1.

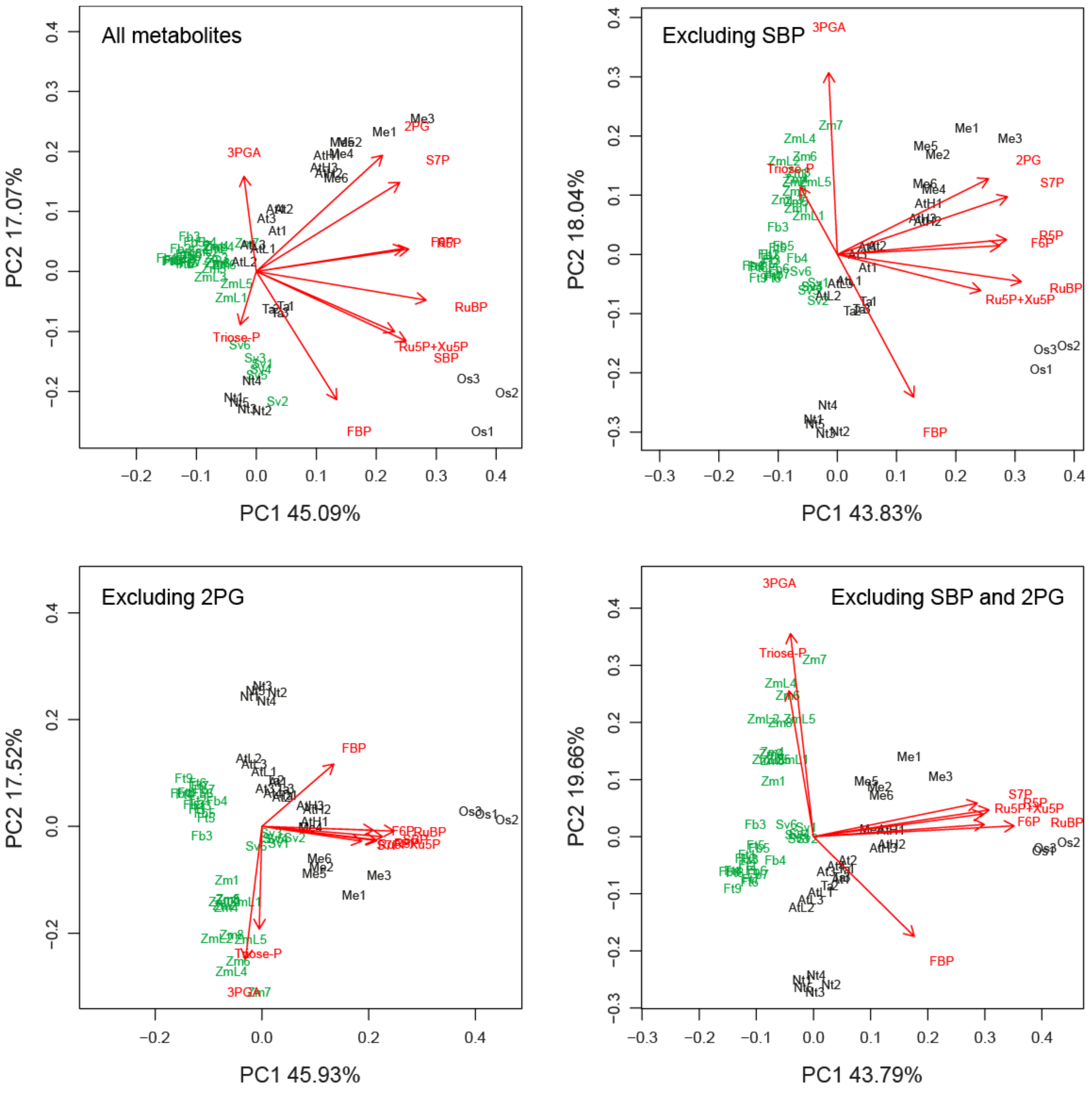

Supplementary Figure S5. PC analyses on all species using metabolite data normalized on total chlorophyll content (supplementary analyses to Figure 4B). Analyses were performed on the entire data set, or excluding 2PG, or excluding SBP, or excluding 2PG and SBP. The distribution of C_4 species (green) and C_3 species (black) is shown on PC1 and PC2 (*Z. mays,* Zm and ZmL*; S. viridis,* Sv; *F. bidentis,* Fb*; F. trinervia,* Ft*; O. sativa,* Os; *T. aestivum,* Ta*; A. thaliana,* AtL, At and AtH*; N. tabacum,* Nt*; M. esculenta,* Me). The loadings of CBC intermediates in PC1 and PC2 are shown in red. The original data are presented in Supplementary Dataset S1.

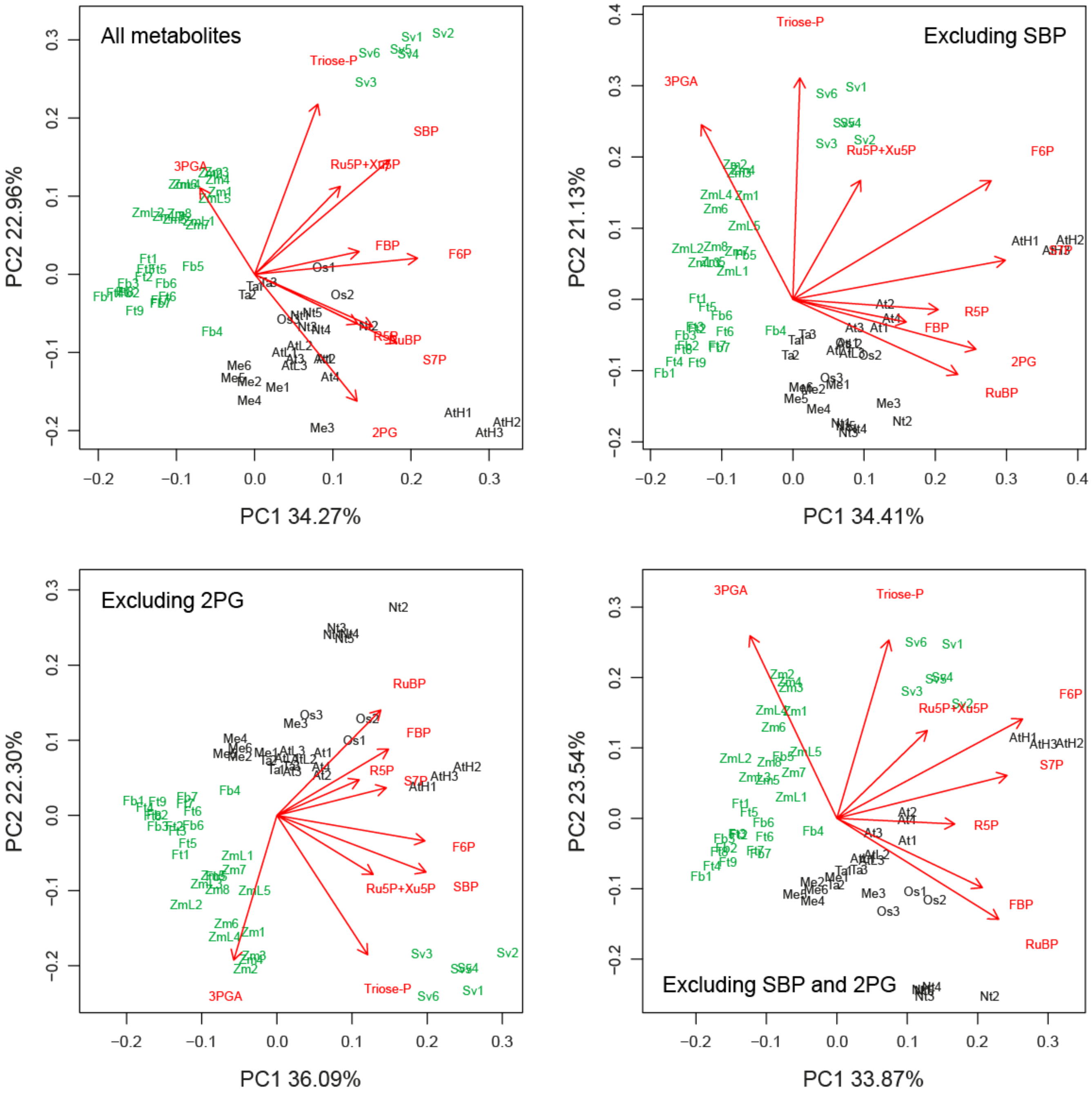

Supplementary Figure S6. PC analyses on all species using metabolite data normalized on protein content (supplementary analyses to Figure 4C). Analyses were performed on the entire data set, or excluding 2PG, or excluding SBP, or excluding 2PG and SBP. The distribution of C_4 species (green) and C_3 species (black) is shown on PC1 and PC2 (*Z. mays,* Zm and ZmL*; S. viridis,* Sv; *F. bidentis,* Fb*; F. trinervia,* Ft*; O. sativa,* Os; *T. aestivum,* Ta*; A. thaliana,* AtL, At and AtH*; N. tabacum,* Nt*; M. esculenta,* Me). The loadings of CBC intermediates in PC1 and PC2 are shown in red. The original data are presented in Supplementary Dataset S1.

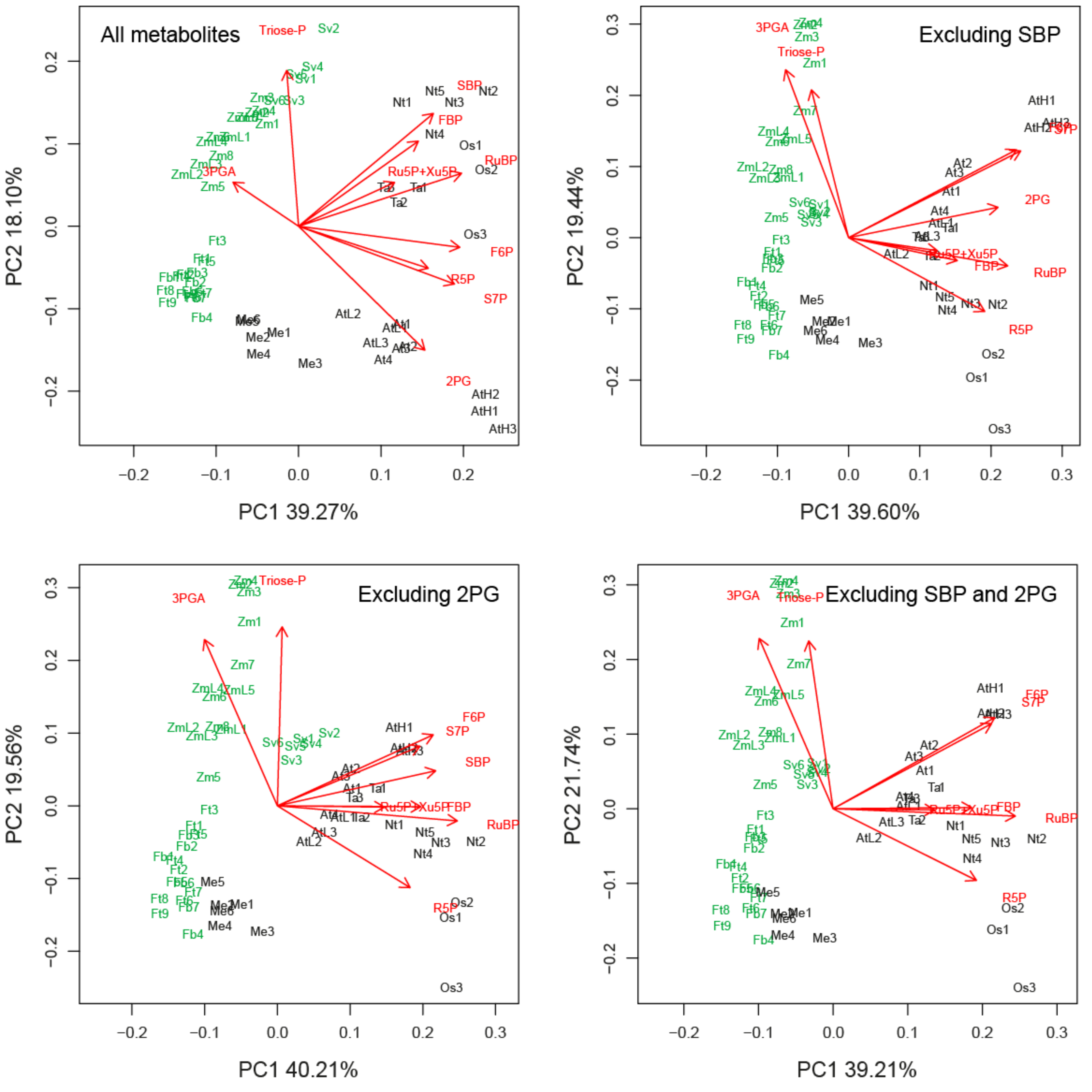

Supplementary Figure S7. PC analyses on all species using a dimensionless data set (supplementary analyses to Figure 4D). Metabolite data were transformed to express them as the C in a given metabolite divided by the total C in all CBC metabolites plus 2PG (i.e. fractional contribution), as explained in the legend of Figure 3. Analyses were performed on the entire data set, or excluding 2PG, or excluding SBP, or excluding 2PG and SBP. The distribution of C_4 species (green) and C_3 species (black) is shown on PC1 and PC2 (*Z. mays,* Zm and ZmL*; S. viridis,* Sv; *F. bidentis,* Fb; *F. trinervia,* Ft; *O. sativa,* Os; *T. aestivum,* Ta*; A. thaliana,* AtL, At and AtH*; N. tabacum,* Nt*; M. esculenta,* Me). The loadings of CBC intermediates in PC1 and PC2 are shown in red. The original data are presented in Supplementary Dataset S1.

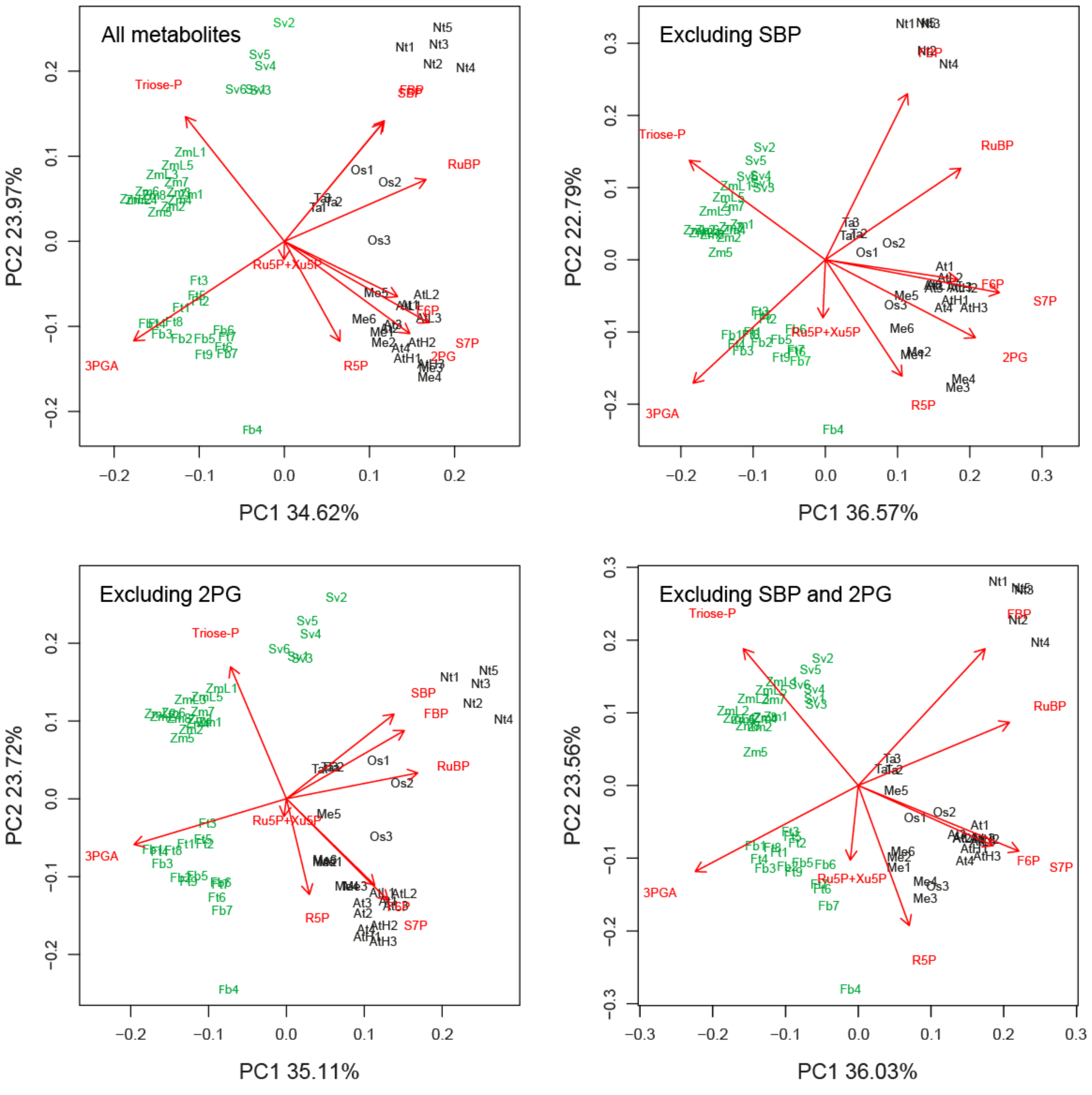

Supplementary Figure S8. PC analyses on C_3 species only, using metabolite data normalized on total chlorophyll content (supplementary analyses to Figure 5A). Analyses were performed on the entire data set, or excluding 2PG, or excluding SBP, or excluding 2PG and SBP. The distribution of C_3 species is shown on PC1 and PC2 (*N. tabacum*, Nt; *O. sativa*, Os; *A. thaliana*, AtL, At and AtH; *T. aestivum*, Ta; *M. esculenta*, Me). The loadings of CBC intermediates in PC1 and PC2 are shown in red. The original data are presented in Supplementary Dataset S1.

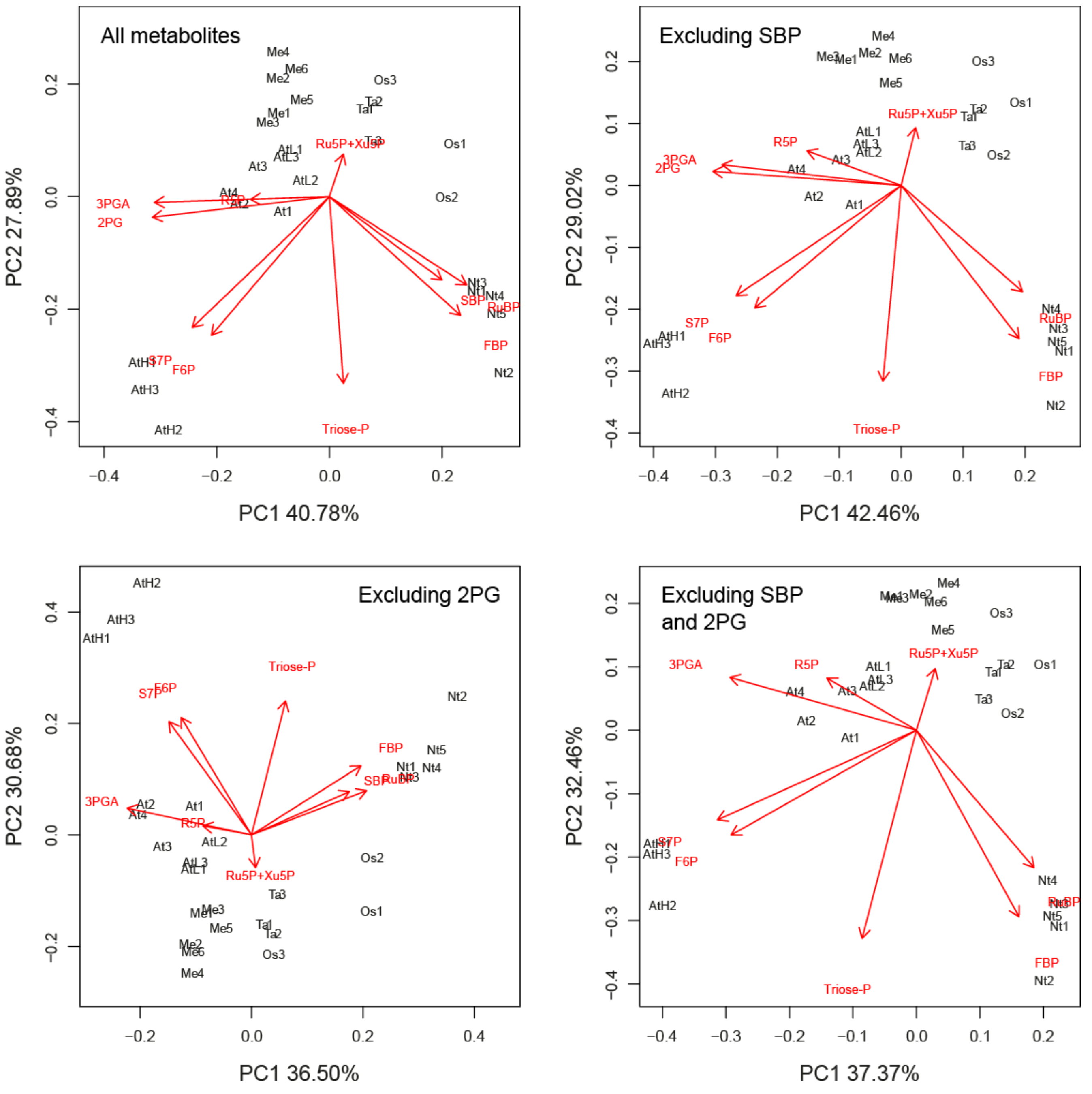

Supplementary Figure S9. PC analyses on C_3 species only, using metabolite data normalized on protein content (supplementary analyses to Figure 5B). Analyses were performed on the entire data set, or excluding 2PG, or excluding SBP, or excluding 2PG and SBP. The distribution of C_3 species is shown on PC1 and PC2 (*N. tabacum*, Nt; *O. sativa*, Os; *A. thaliana*, AtL, At and AtH; *T. aestivum*, Ta; *M. esculenta*, Me). The loadings of CBC intermediates in PC1 and PC2 are shown in red. The original data are presented in Supplementary Dataset S1.

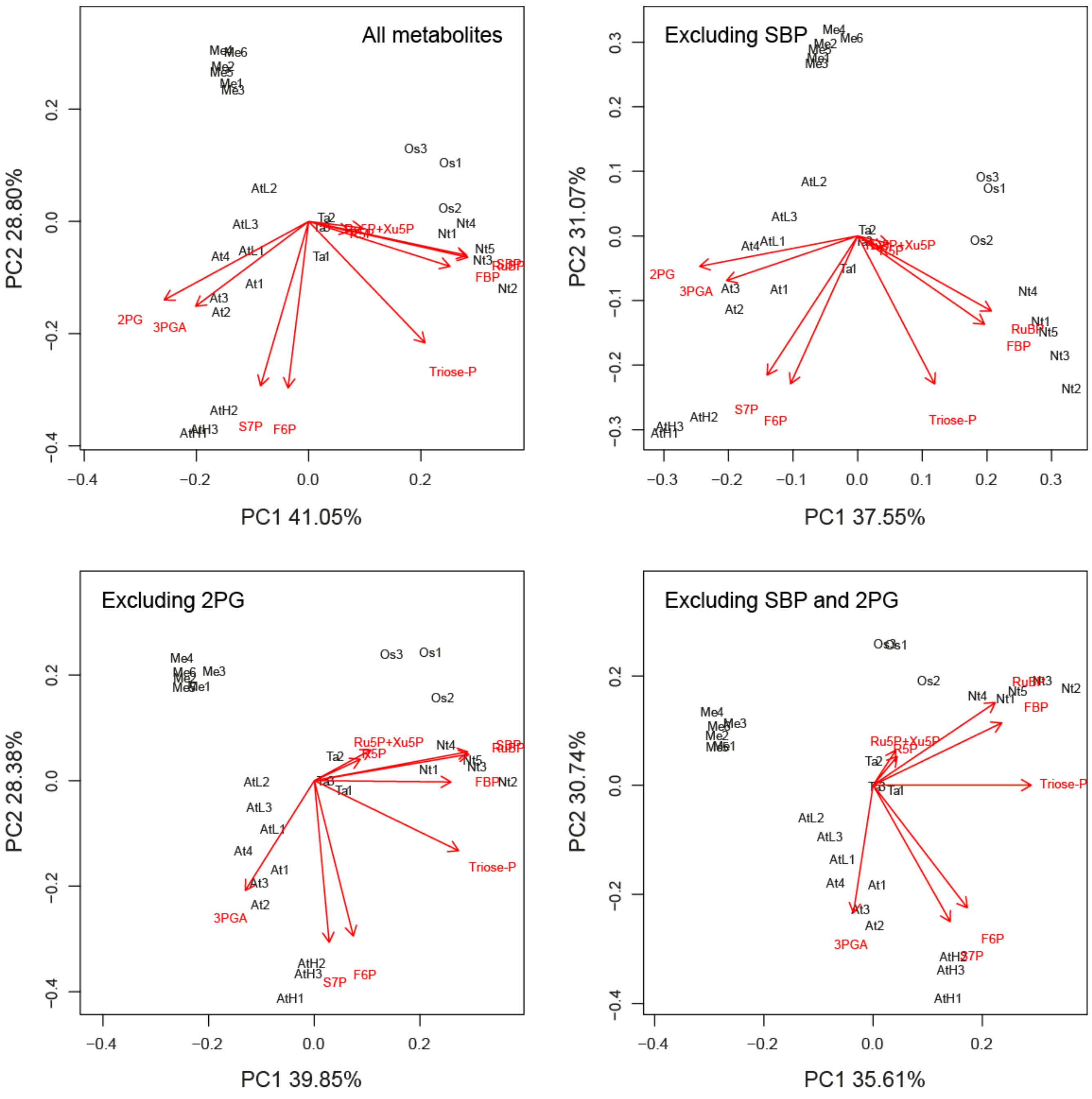

Supplementary Figure S10. PC analyses on C_3 species only, using a dimensionless data set (supplementary analyses to Figure 5C). Analyses were performed on the entire data set, or excluding 2PG, or excluding SBP, or excluding 2PG and SBP. The distribution of C_3 species is shown on PC1 and PC2 (*N. tabacum*, Nt; *O. sativa*, Os; *A. thaliana*, AtL, At and AtH; *T. aestivum*, Ta; *M. esculenta*, Me). The loadings of CBC intermediates in PC1 and PC2 are shown in red. The original data are presented in Supplementary Dataset S1.

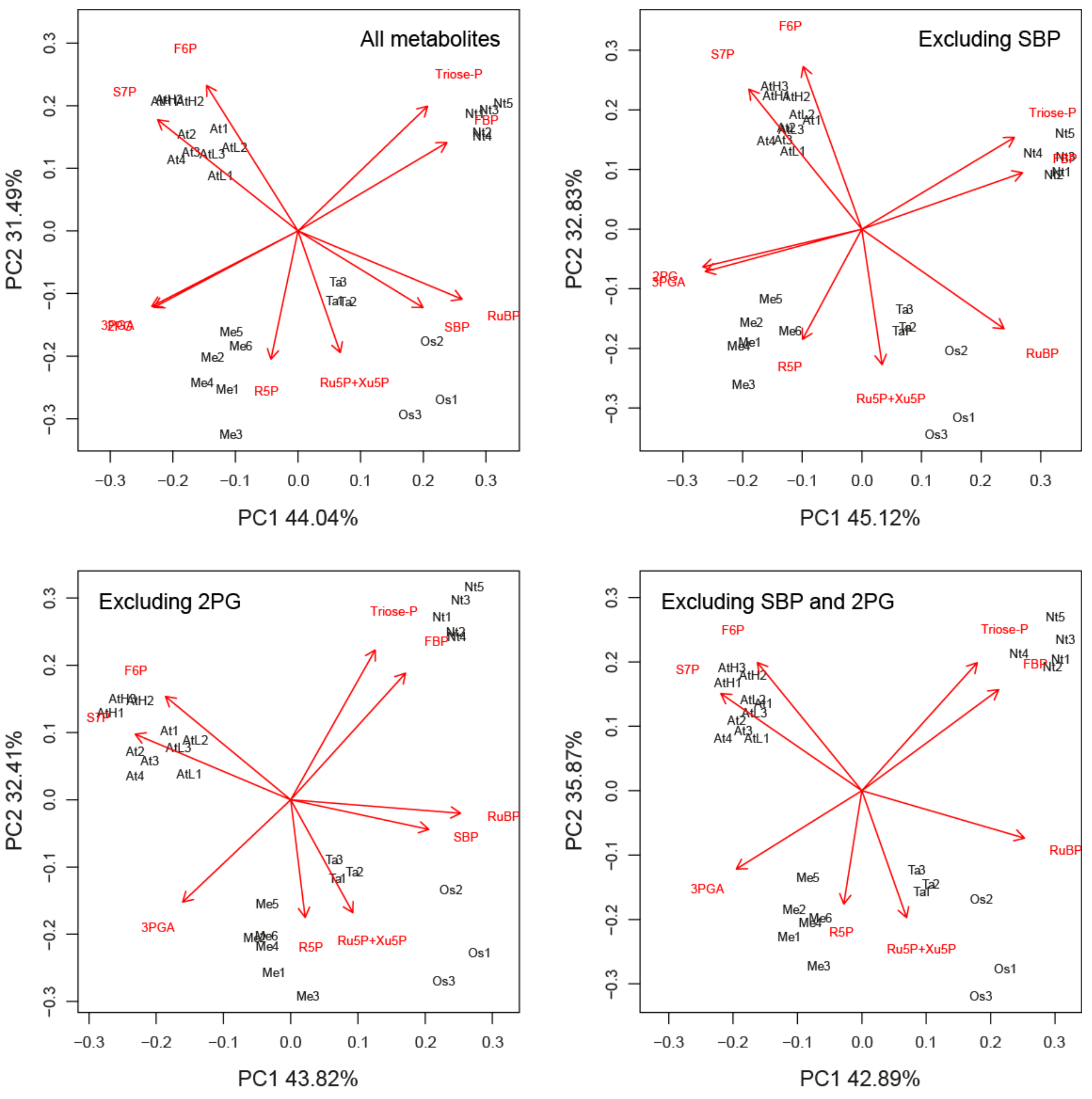

Supplementary Figure S11. Correlation between levels of CBC metabolites, with metabolites shown in a fixed order reflecting the reaction sequence in the CBC. The correlations were performed as described for Figure 7, using all individual samples from a dimensionless data set generated as described for Figure 4D, except that metabolites were not clustered. Correlation values are given in the figure panels and indicated by a heat map. Non-significant correlations ($p \geq 0.05$; two-sided Student's t-test) are set as zero. Metabolite pairs that are linked by irreversible reactions are indicated by a black box. **(A)** All species, **(B)** only C_4 species, and **(C)** only C_3 species.

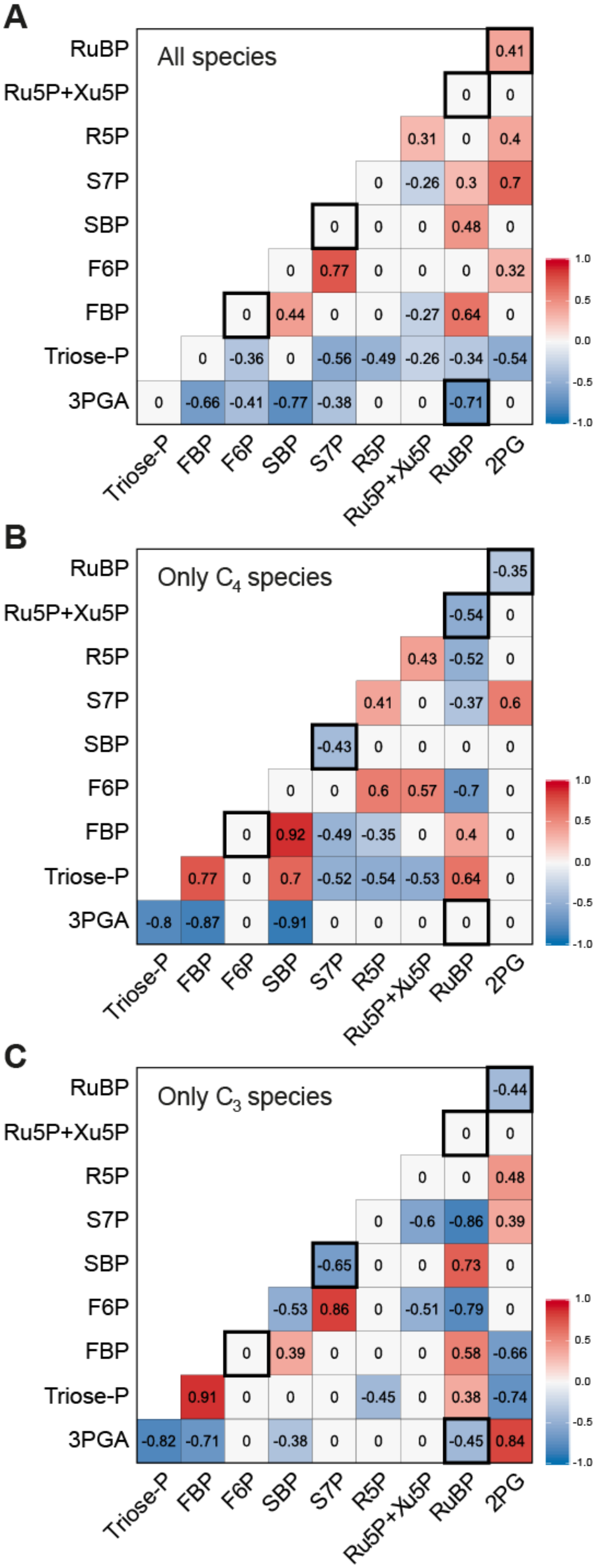

Supplement: Supplementary Figure S1 [file erz051_suppl_supplementary_figure_s1.pdf]
